# Genomic evolution towards azole resistance in *Candida glabrata* clinical isolates unveils the importance of CgHxt4/6/7 in azole accumulation

**DOI:** 10.1038/s42003-022-04087-0

**Published:** 2022-10-21

**Authors:** Mónica Galocha, Romeu Viana, Pedro Pais, Ana Silva-Dias, Mafalda Cavalheiro, Isabel M. Miranda, Mieke Van Ende, Caio S. Souza, Catarina Costa, Joana Branco, Cláudio M. Soares, Patrick Van Dijck, Acácio G. Rodrigues, Miguel C. Teixeira

**Affiliations:** 1 Department of Bioengineering, Instituto Superior Técnico, Universidade de Lisboa, Lisbon, Portugal; 2 iBB - Institute for Bioengineering and Biosciences, Biological Sciences Research Group, Instituto Superior Técnico, Lisbon, Portugal; 3 Associate Laboratory i4HB—Institute for Health and Bioeconomy at Instituto Superior Técnico, Universidade de Lisboa, Lisbon, Portugal; 4 Department of Microbiology, Faculty of Medicine, University of Porto, Porto, Portugal; 5 CINTESIS - Center for Health Technology and Services Research, Faculty of Medicine, University of Porto, Porto, Portugal; 6 Cardiovascular R&D Center, Faculty of Medicine, University of Porto, Porto, Portugal; 7 Laboratory of Molecular Cell Biology, Department of Biology, Institute of Botany and Microbiology, KU Leuven, Leuven, Belgium; 8 VIB-KU Leuven Center for Microbiology, Leuven, Belgium; 9 Instituto de Tecnologia Química e Biológica António Xavier, Universidade Nova de Lisboa, Oeiras, Portugal

**Keywords:** Pathogenesis, Microbiology

## Abstract

The increasing prevalence of candidosis caused by *Candida glabrata* is related to its ability to acquire azole resistance. Although azole resistance mechanisms are well known, the mechanisms for azole import into fungal cells have remained obscure. In this work, we have characterized two hexose transporters in *C. glabrata* and further investigate their role as potential azole importers. Three azole susceptible *C. glabrata* clinical isolates were evolved towards azole resistance and the acquired resistance phenotype was found to be independent of *CgPDR1* or *CgERG11* mutations. Through whole-genome sequencing, *CgHXT4/6/7* was found to be mutated in the three evolved strains, when compared to their susceptible parents. CgHxt4/6/7 and the 96% identical CgHxt6/7 were found to confer azole susceptibility and increase azole accumulation in *C. glabrata* cells, strikingly rescuing the susceptibility phenotype imposed by *CgPDR1* deletion, while the identified loss-of-function mutation in *CgHXT4/6/7*, leads to increased azole resistance. In silico docking analysis shows that azoles display a strong predicted affinity for the glucose binding site of CgHxt4/6/7. Altogether, we hypothesize that hexose transporters, such as CgHxt4/6/7 and CgHxt6/7, may constitute a family of azole importers, involved in clinical drug resistance in fungal pathogens, and constituting promising targets for improved antifungal therapy.

## Abstract (web summary)

Mutations in the hexose transporter, CgHXT4/6/7, contribute to increased antifungal (azole) resistance in the fungal pathogen, *Candida glabrata*, potentially by influencing azole accumulation.

## Introduction

*Candida* species are one of the most common cause of fungemia in humans, responsible for >400,000 life-threatening infections yearly worldwide with an associated 46–75% mortality rate^1^. Clinical isolates resistant to all three classes of antifungals available have been found in hospitalized patients^2^. *C. glabrata*, particularly, is a prominent cause of invasive candidosis, best known for its ability to overcome azole antifungal therapy^3–6^.

Azoles act by inhibiting the 14α-demethylase Erg11 in the ergosterol biosynthesis pathway, causing the accumulation of a toxic sterol that permeabilizes the plasma membrane^7^. These drugs have been used for decades as the standard treatment against candidosis and are still the only oral treatment option. Instead of developing new drugs to overcome antifungal resistance, a process which is both very costly and time-consuming, therapeutic strategies can be improved by enhancing the efficacy of existing drugs, such as azoles. Therefore, it is worth identifying the genes and cellular pathways involved in the development of drug resistance^8^.

The main mechanism of acquired azole resistance is the up-regulation of ABC (ATP-binding Cassette) drug transporters, mainly CgCdr1^9–11^, and, to a lower extent, of the major facilitator superfamily (MFS) drug:H + antiporters^12–19^, which catalyse the efflux of azoles preventing their intracellular accumulation. Generally, the constitutive up-regulation of the ABC efflux pumps is due to the emergence of gain-of-function (GOF) mutations in the *CgPDR1* gene, encoding the key regulator of multidrug resistance in *C. glabrata*^4, 20^. In contrast to what is observed in *C. albicans*, several studies suggest that mutations in *CgERG11* is not a relevant mechanism of acquired azole resistance in *C. glabrata* clinical isolates^5, 9, 21^. Although *CgPDR1* GOF mutations are considered the major mechanism of clinical acquisition of azole resistance, several resistant clinical isolates do not to display any of the known mechanisms of resistance^22, 23^, suggesting the presence of yet unknown alternative paths to acquired azole resistance in *C. glabrata*.

In this study, prolonged exposure to posaconazole was used to induce in vitro *C. glabrata* azole susceptible clinical isolates to acquire azole resistance. The underlying molecular mechanisms were evaluated through whole-genome sequencing. Genes found to exhibit non-synonymous single-nucleotide polymorphisms (nsSNPs) in the resistant strains, when compared to their susceptible counterparts, were identified and evaluated for a possible role in azole resistance. The putative hexose transporter *CgHXT4/6/7* (ORF *CAGL0A02233g*) stood out in the analysis, being hypothesized to work as an azole importer. Although little is known about how these drugs enter pathogenic fungal cells to exert their antifungal action^24^, it was demonstrated that azoles are imported by facilitated diffusion rather than passive diffusion or ATP-dependent import^25^. Moreover, fluconazole import differs among *C. albicans* resistant clinical isolates, which suggests that altered facilitated diffusion is possibly a previously uncharacterized mechanism of resistance to azole drugs^25^.

In this context, the role of CgHxt4/6/7 and of the 96% identical CgHxt6/7 (ORF *CAGL0A02211g*)^26^ as hexose transporters was confirmed, while their role as potential azole importers was further explored leading to the characterization of an alternative mechanism of acquired azole drug resistance in *C. glabrata*.

## Results

### In vitro evolution towards posaconazole resistance in *C. glabrata* clinical isolates

Four isolates, previously found to display azole susceptibility^27^, the CBS138 reference strain and three clinical isolates (040, 044 and OL152), were subjected to an in vitro directed evolution approach, to generate azole-resistant populations. After prolonged exposure to a therapeutic plasma concentration of the triazole posaconazole (1 mg L^−1^), the CBS138, 040 and OL152 isolates evolved towards posaconazole, fluconazole and clotrimazole resistance following 10 days of incubation, while the 044 isolate acquired resistance to clotrimazole, fluconazole, voriconazole and posaconazole after 25 days of incubation (Supplementary Table 1). Interestingly, MIC_50_ levels for voriconazole never reached resistance levels for CBS138, 040 and OL152 isolates (Supplementary Table 1). The evolved multiazole-resistant strains were denominated CBS138_Psc, 040_Psc, 044_Psc and OL152_Psc. The resistance pattern remained stable in all the evolved resistant isolates following sub-culture in drug-free medium for up to 30 days.

### Acquisition of azole resistance can be independent of mutations in *CgPDR1* or *CgERG11*

To understand whether the evolved resistance phenotype relied on the major mechanism of acquired azole resistance, the acquisition of Pdr1 GOF mutations, the transcription factor encoding gene *CgPDR1* was sequenced in the four evolved resistant strains (CBS138_Psc, 040_Psc, 044_Psc and OL152_Psc). Remarkably, no sequence changes were detected in 040_Psc, 044_Psc or OL152_Psc and so the molecular basis underlying the development of azole resistance on these three evolved strains remained to be clarified. In the azole-resistant strain derived from the CBS138 strain, CBS138_Psc, a mutation in the *CgPDR1* gene sequence was found. In this single case, indeed a non-synonymous mutation leading to a Trp297Leu substitution was observed, which corresponds to a previously characterized GOF mutation in *CgPDR1*^23^. This mutation is likely underlying the observed resistance phenotype in the evolved strain, which was therefore excluded from further analysis. Furthermore, no changes in the sequence or expression (Supplementary Fig. 1) of the drug target, CgErg11, were found in the four evolved strains, reinforcing the assumption that alteration of the drug target is not a usual mechanism of acquired azole resistance in *C. glabrata* clinical isolates. Potential resistance-related mutations in the promoter regions of both *CgPDR1* and *CgERG11* were also not found (Supplementary Data 1).

### Identification of nsSNPs acquired during posaconazole exposure

In the search for insights into the mechanism(s) underlying the development of azole resistance in the evolved strains 040_Psc, 044_Psc and OL152_Psc, their genomes were sequenced and compared to that of the corresponding susceptible parents 040, 044 and OL152, respectively (Supplementary Data 2; Supplementary Data 3). 40, 70 and 103 nsSNPs were registered for the 040_Psc, 044_Psc and OL152_Psc strains, respectively, when compared to the susceptible parents (Supplementary Fig. 2a). These nsSNPs were found to affect 19, 22 and 28 genes in 040_Psc, 044_Psc and OL152_Psc, respectively (Supplementary Fig. 2b), which were grouped based on their biological function (Supplementary Table 2). Most of the mutated genes were found to encode adhesins or adhesin-like proteins, specifically 70%, 92% and 70% of the mutated genes in the 040_Psc, 044_Psc and OL152_Psc strains, respectively. This is likely a direct consequence of adhesin encoding genes being mostly located in sub-telomeric regions and including tandem-repeat regions, which favours increased random mutation rates. Nonetheless, it is interesting to point out that at least the *CgEPA3* adhesin has been implicated in azole resistance^28^.

Despite no common nsSNPs selected during prolonged posaconazole exposure among the 040_Psc, 044_Psc and OL152_Psc strains (Supplementary Fig. 2a) were registered, 5 genes were found to display non-synonymous mutations in the three resistant strains, when compared to the susceptible counterparts. These genes included 4 adhesin-like proteins, including *CgEPA3*, plus the putative hexose transporter *CgHXT4/6/7* (Supplementary Fig. 2b), which was selected for further analysis.

### CgHxt4/6/7 and CgHxt6/7 are plasma membrane hexose transporters

**Fig. 1:**
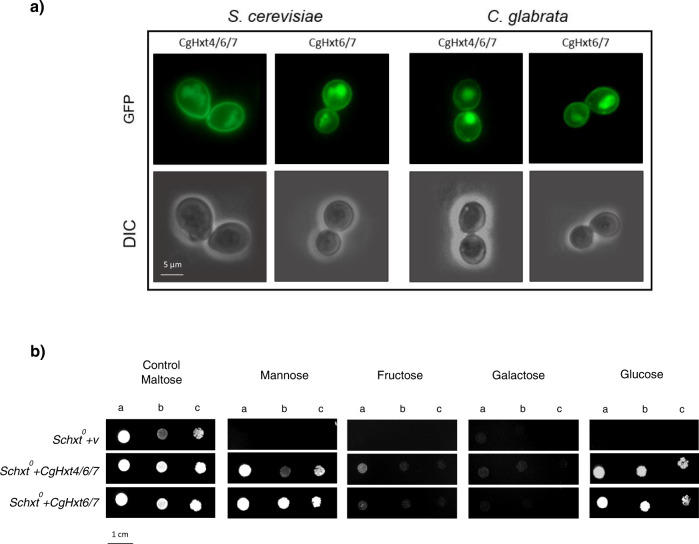
CgHxt4/6/7 and CgHxt6/7 are plasma membrane hexose transporters. **a** Fluorescence of exponential phase BY4741 *S. cerevisiae* cells harbouring the plasmids pGREG576_TEF_*CgHXT4/6/7* (CgHxt4/6/7_GFP) or pGREG576_TEF_*CgHXT6/7* (CgHxt6/7_GFP), and KUE100::URA- *C. glabrata* cells harbouring the plasmids pGREG576_PDC1_*CgHXT4/6/7* (CgHxt4/6/7_GFP) or pGREG576_PDC1_*CgHXT6/7* (CgHxt6/7_GFP), after 6 h of constitutive recombinant protein production. **b** Comparison of spot growth assays of the EBY.VW4000 (*Schxt*^*0*^) strain harbouring the pGREG567_TEF vector or the pGREG576_TEF_*CgHXT4/6/7* or pGREG576_TEF_*CgHXT6/7* expression plasmids in the presence of different indicated hexoses as carbon sources. The inocula were prepared as described in the Methods section. Cell suspensions used to prepare the spots were 1:5 (b) and 1:25 (c) dilutions of the cell suspensions used in (a). The displayed images are representative of at least three independent experiments.

The subcellular localization of CgHxt4/6/7 and of the 96% identical CgHxt6/7 was inspected by fluorescence microscopy in both *C. glabrata* and *S. cerevisiae* cells. As expected, the CgHxt4/6/7_GFP and the CgHxt6/7_GFP fusion proteins were found to be predominantly localized to the cell periphery, which is consistent with its predicted plasma membrane localization and transporter function, in both *C. glabrata* and *S. cerevisiae* cells (Fig. 1a).

The ability of the putative hexose transporters CgHxt4/6/7 and CgHxt6/7 to import the hexoses glucose, mannose, fructose and galactose into yeast cells was assessed using the hexose transporter-null *S. cerevisiae* strain (*Schxt*^*0*^), which grows normally on maltose as carbon source^29^. The *Schxt*^*0*^ strain is unable to grow on glucose, mannose or fructose as sole carbon sources and grows very poorly on galactose^29^. Results demonstrate that *Schxt*^*0*^ cells expressing either CgHxt4/6/7 or CgHxt6/7 were able to grow on glucose and mannose and slightly on fructose as carbon sources, while cells harbouring the cloning vector were not (Fig. 1b). Together, these results demonstrate that CgHxt4/6/7 and CgHxt6/7 are plasma membrane hexose transporters, which was expected considering the role of their *S. cerevisiae* orthologues ScHxt6 and ScHxt7, respectively^30^.

### *CgHXT4/6/7* and *CgHXT6/7* promote azole drug susceptibility in *C. glabrata*, probably by facilitating azole entrance into fungal cells

**Fig. 2:**
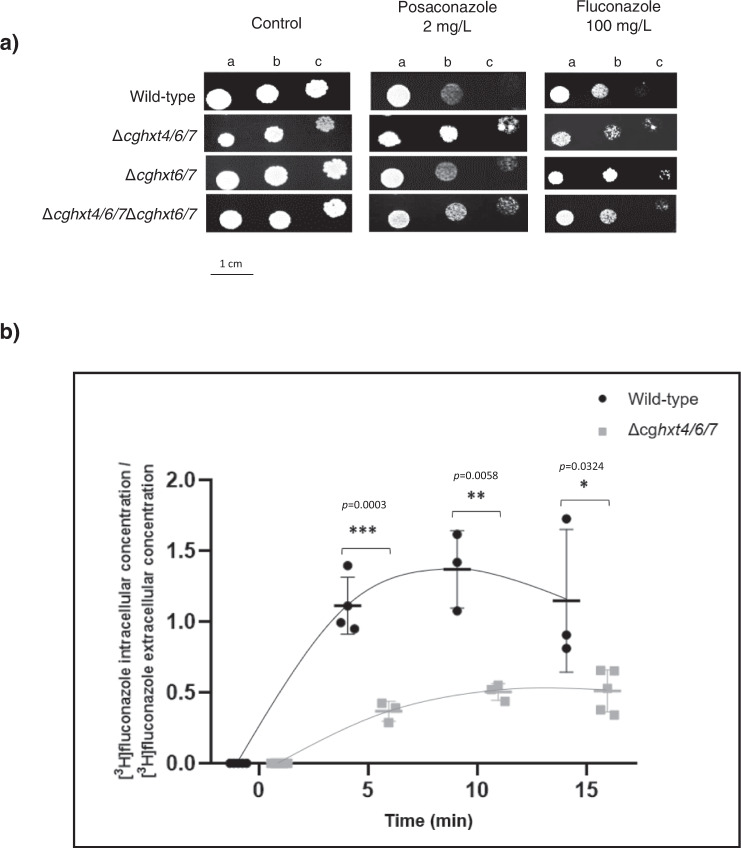
CgHxt4/6/7 and CgHxt6/7 promote azole susceptibility in *C. glabrata*. **a** Comparison of the susceptibility to antifungal azole drugs, at the indicated concentrations, of the *C. glabrata* KEU100 wild-type strain or derived KUE100_Δ*cghxt4/6/7*, KUE100_Δ*cghxt6/7* or KUE100_Δ*cghxt4/6/7*Δ*cghxt6/7* mutant cells on YPD agar plates by spot assays. The inoculum was prepared as described in the Methods section. Cell suspensions used to prepare the spots correspond to 1:5 (b) and 1:25 (c) dilutions of the cell suspensions used in (a). The displayed images are representative of at least three independent experiments. **b** Time-course accumulation ratio of [^3^H]-Fluconazole in non-adapted KUE100 wild-type cells (black circles) or derived KUE100_Δ*cghxt4/6/7* mutant cells (grey squares) during cultivation in liquid YPD medium in the presence of unlabelled fluconazole. The accumulation ratio values are averages of, at least, *n* = 3 independent experiments. Error bars represent the corresponding standard deviations. Significance levels are attributed as follows: *p value < 0.05; **p value < 0.01; ***p value < 0.001.

Since the so far uncharacterized hexose transporter encoding gene *CgHXT4/6/7* stood out in the genome-wide analysis of the evolved resistant strains 040_Psc, 044_Psc and OL152_Psc, the potential impact of this transporter in *C. glabrata* tolerance to azoles was further evaluated, along with its close homologue *CgHXT6/7*. By comparing the susceptibility of wild-type, single deletion strains (*Δcghxt4/6/7* and *Δcghxt6/7*) and double deletion strain (*Δcghxt4/6/7Δcghxt6/7*), the absence of either *HXT* gene was found to increase *C. glabrata* resistance towards fluconazole and posaconazole (Fig. 2a). Accordingly, MIC_50_ values for fluconazole and posaconazole were found to be lower for the wild-type strain (16 and 0.5 mg L^−1^, respectively) when compared to the single deletion strains *Δcghxt4/6/7* (32 and 1 mg L^−1^, respectively) or *Δcghxt6/7* (32 and 1 mg L^−1^, respectively), and to the double deletion strain *Δcghxt4/6/7Δcghxt6/7* (64 and 2 mg L^−1^, respectively).

**Fig. 3:**
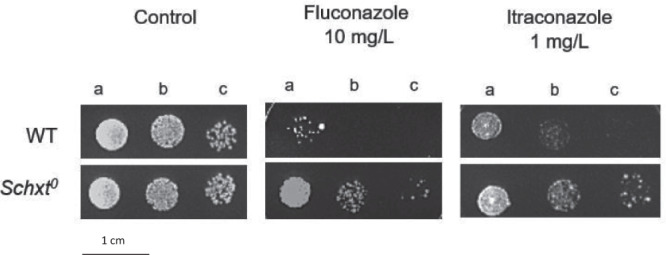
*HXT* genes play a role in yeast susceptibility towards azole antifungals. Comparison of the susceptibility to azole drugs, at the indicated concentrations, of the CEN.PK2-1C *S. cerevisiae* wild-type strain and the derived EBY.VW4000 (*Schxt*^*0*^) strain on YPM agar plates by spot assays. The inoculum was prepared as described in the Methods section. Cell suspensions used to prepare the spots correspond to 1:5 (b) and 1:25 (c) dilutions of the cell suspensions used in (a). The displayed images are representative of at least three independent experiments.

The results obtained for either CgHxt4/6/7 or CgHxt6/7 are consistent, but moderate, likely because 10 other hexose transporters are predicted to be encoded by the *C. glabrata* genome, potentially displaying functional overlap. In fact, the double deletion strain *Δcghxt4/6/7Δcghxt6/7* was found to display a fourfold increase in the MIC_50_ for both fluconazole and posaconazole, comparing to the wild-type. To assess the concept of functional overlap, the hexose transporter-null *Schxt*^*0*^ mutant was compared with the corresponding parental strain in terms of azole susceptibility. Hexose transporter-null *Schxt*^*0*^ cells are indeed less susceptible to the azoles tested (Fig. 3). Although this finding needs to be further assessed in *C. glabrata*, this result strongly supports the hypothesis that several of the *HXT* transporters might play a key role as azole importers, therefore contributing to azole susceptibility in fungal cells. Accordingly, the accumulation of [^3^H]-labelled fluconazole was evaluated in the single deletion strain ∆*cghxt4/6/7* comparing to the wild-type. Under these conditions, cells lacking *CgHXT4/6/7* accumulate significantly less fluconazole than the parental wild-type cells (Fig. 2b). Moreover, the same effect was observed when measuring the accumulation of the [^3^H]-labelled imidazole clotrimazole in cells lacking *CgHXT4/6/7*, comparing to wild-type cells (Supplementary Fig. 3), further supporting the hypothesis that CgHxt4/6/7 affects azole uptake in *C. glabrata*.

### Deletion of *CgHXT4/6/7* in the 040 susceptible clinical isolate promotes azole resistance

**Fig. 4:**
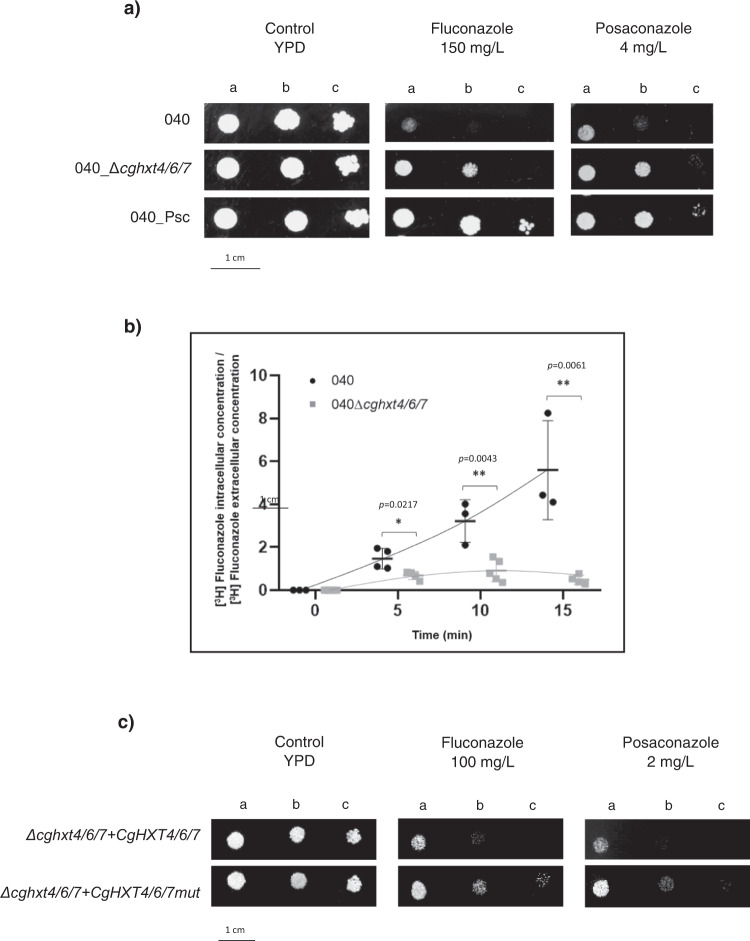
CgHxt4/6/7 promotes azole susceptibility in *C. glabrata* 040 clinical isolate by mediating azoles uptake. **a** Comparison of the susceptibility to antifungal azole drugs, at the indicated concentrations, of the *C. glabrata* 040 clinical isolate, the derived mutant strain 040_Δ*cghxt4/6/7* and the evolved resistant strain 040_Psc on YPD agar plates by spot assays. The inoculum was prepared as described in the Methods section. Cell suspensions used to prepare the spots correspond to 1:5 (b) and 1:25 (c) dilutions of the cell suspensions used in (a). The displayed images are representative of at least three independent experiments. **b** Time-course accumulation ratio of [^3^H]-Fluconazole in non-adapted (black circles) 040 or derived (grey squares) 040_Δ*cghxt4/6/7* mutant cells during cultivation in liquid YPD medium in the presence of unlabelled fluconazole. The accumulation ratio values are averages of, at least, *n* = 3 independent experiments. Error bars represent the corresponding standard deviations. Significance levels are attributed as follows: *p value < 0.05; **p value < 0.01. **c** Comparison of the susceptibility to antifungal azole drugs, at the indicated concentrations, of the *C. glabrata* KUE100::URA-_Δ*cghxt4/6/7* mutant strain harbouring the pGREG576_PDC1_*CgHXT4/6/7* or pGREG576_PDC1_mut_*CgHXT6/7* on BM agar plates (without uracil, for plasmid maintenance) by spot assays. The inocula were prepared as described in the Methods section. Cell suspensions used to prepare the spots were 1:5 (b) and 1:25 (c) dilutions of the cell suspensions used in (a). The displayed images are representative of at least three independent experiments.

The relevance of CgHxt4/6/7 in the azole resistance phenotype developed by the 040 clinical isolate when exposed to posaconazole was further evaluated. Susceptibility testing demonstrates that the deletion of *CgHXT4/6/7* in the 040 isolate decreases its susceptibility towards posaconazole and fluconazole (Fig. 4a). MIC_50_ values of fluconazole and posaconazole were found to be lower for the azole susceptible isolate 040 (16 and 0.125 mg L^−1^, respectively) when compared to the derived mutant strain 040_∆*cghxt4/6/7* (32 and 2 mg L^−1^, respectively), while for the evolved 040_Psc resistant strain the values were even higher (≥64 and ≥8 mg L^−1^, respectively). Accordingly, the accumulation of [^3^H]-labelled fluconazole was shown to be significantly lower in the 040_∆*cghxt4/6/7* strain when comparing to the susceptible parent isolate 040 (Fig. 4b). Although it is noteworthy that the overall accumulation of [^3^H]-labelled fluconazole appears to be higher in the 040 clinical isolate, when compared to the KUE100 lab strain, likely due to extensive genomic background differences, the impact of *CgHXT4/6/7* expression in azole accumulation is clearly strain-independent. Additionally, the G361A missense mutation found in the *CgHXT4/6/7* gene from the evolved 040_Psc strain, resulting in a Val121ILe amino acid residue substitution, was evaluated for its impact in the acquired azole-resistant phenotype (Fig. 4c). In that sense, the pGREG576_PDC1_mut_*CgHXT4/6/7* plasmid was generated by site-directed mutagenesis. The introduction of the pGREG576_PDC1_mut_*CgHXT4/6/7* plasmid in the single deletion mutant strain ∆*cghxt4/6/7* did not affect the MIC_50_ values for fluconazole (MIC_50_ = 32mg L-1) and posaconazole (MIC_50_ = 1 mg L^−1^), while its complementation with the pGREG576_PDC1_*CgHXT4/6/7* plasmid, expressing the wild-type CgHxt4/6/7 transporter, resulted in promoted susceptibility of the ∆*cghxt4/6/7* strain, e.g. displaying lower MIC_50_ values for fluconazole (MIC_50_ = 16 mg L^−1^) and posaconazole (MIC_50_ = 0.5 mg L^−1^) (Fig. 4c).

Altogether, these results strongly suggest that *CgHXT4/6/7* plays a role in *C. glabrata* azole drug susceptibility and that the nsSNP selected in *CgHXT4/6/7* of the 040 isolate during the in vitro evolution experiment impaired of the transporter function, contributing to the final resistant phenotype observed in the evolved strain 040_Psc.

### Deletion of either *CgHXT4/6/7* or *CgHXT6/7* rescues the susceptibility phenotype imposed by the absence of the drug resistance regulator *CgPDR1*

**Fig. 5:**
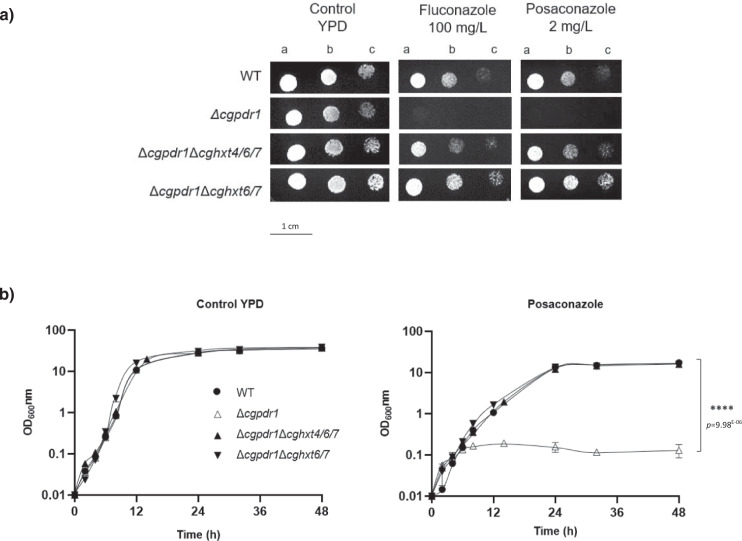
Deletion of either *CgHXT4/6/7* or *CgHXT6/7* in a ∆*cgpdr1* background rescues the azole susceptible phenotype imposed by the absence of CgPdr1. **a** Comparison of the susceptibility to antifungal azole drugs, at the indicated concentrations, of the KUE100 *C. glabrata* wild-type strain and the derived mutant strains KUE100_Δ*cgpdr1*, KUE100_Δ*cgpdr1*Δ*cghxt4/6/7*, and KUE100_Δ*cgpdr1*Δ*cghxt6/7* on YPD agar plates by spot assays. The inoculum was prepared as described in the “Methods” section. Cell suspensions used to prepare the spots correspond to 1:5 (b) and 1:25 (c) dilutions of the cell suspensions used in (a). The displayed images are representative of, at least, *n* = 3 independent experiments. **b** Comparison of the growth curves of the *C. glabrata* wild-type strain KUE100 and the derived mutant strains KUE100_Δ*cgpdr1*, KUE100_Δ*cgpdr1*Δ*cghxt4/6/7*, and KUE100_Δ*cgpdr1*Δ*cghxt6/7*, in liquid YPD, in the absence or presence of 4 mg/L posaconazole, measured in terms of variation in OD_600_. The displayed growth curves are representative, at least, *n* = 3 independent experiments. Error bars represent the corresponding standard deviation. Significance levels are attributed as follows: ****p value <0.0001.

CgPdr1 is the major regulator of azole resistance in *C. glabrata*, controlling the overexpression of azole drug efflux pumps. Consequently, cells lacking the *CgPDR1* gene are highly susceptible to azoles. Surprisingly, the deletion of either *CgHXT4/6/7* or *CgHXT6/7* was found to rescue the azole susceptibility phenotype of *CgPDR1* disrupted cells (Fig. 5a). Strikingly, MIC_50_ values for fluconazole and posaconazole were found to be similar for the wild-type strain (16 and 0.5 mg L^−1^, respectively) and for the double deletion strains ∆*cgpdr1*∆*cghxt4/6/7* or ∆*cgpdr1*∆*cghxt6/7* (16 and 0.5 mg L^−1^, respectively), while for the ∆*cgpdr1* strain the MIC_50_ values were found to be 4–fold lower for fluconazole (4 mg L^−1^) and 32-fold lower for posaconazole (0.015625 mg L^−1^). Accordingly, growth curves obtained in the presence of inhibitory concentrations of posaconazole (4 mg L^−1^) demonstrate that the deletion of either CgHxt4/6/7 or CgHxt6/7 in the ∆*cgpdr1* strain fully rescues its azole susceptibility phenotype (Fig. 5b).

These results further support the hypothesis that these putative hexose transporters play a crucial role in *C. glabrata* azole drug susceptibility, probably by facilitating azole entrance into fungal cells. More importantly, this points out the critical relevance of these transporters in azole drug mode of action and in the consequent mechanism of drug resistance.

### CgHxt4/6/7 is predicted to display high affinity for fluconazole binding

**Fig. 6:**
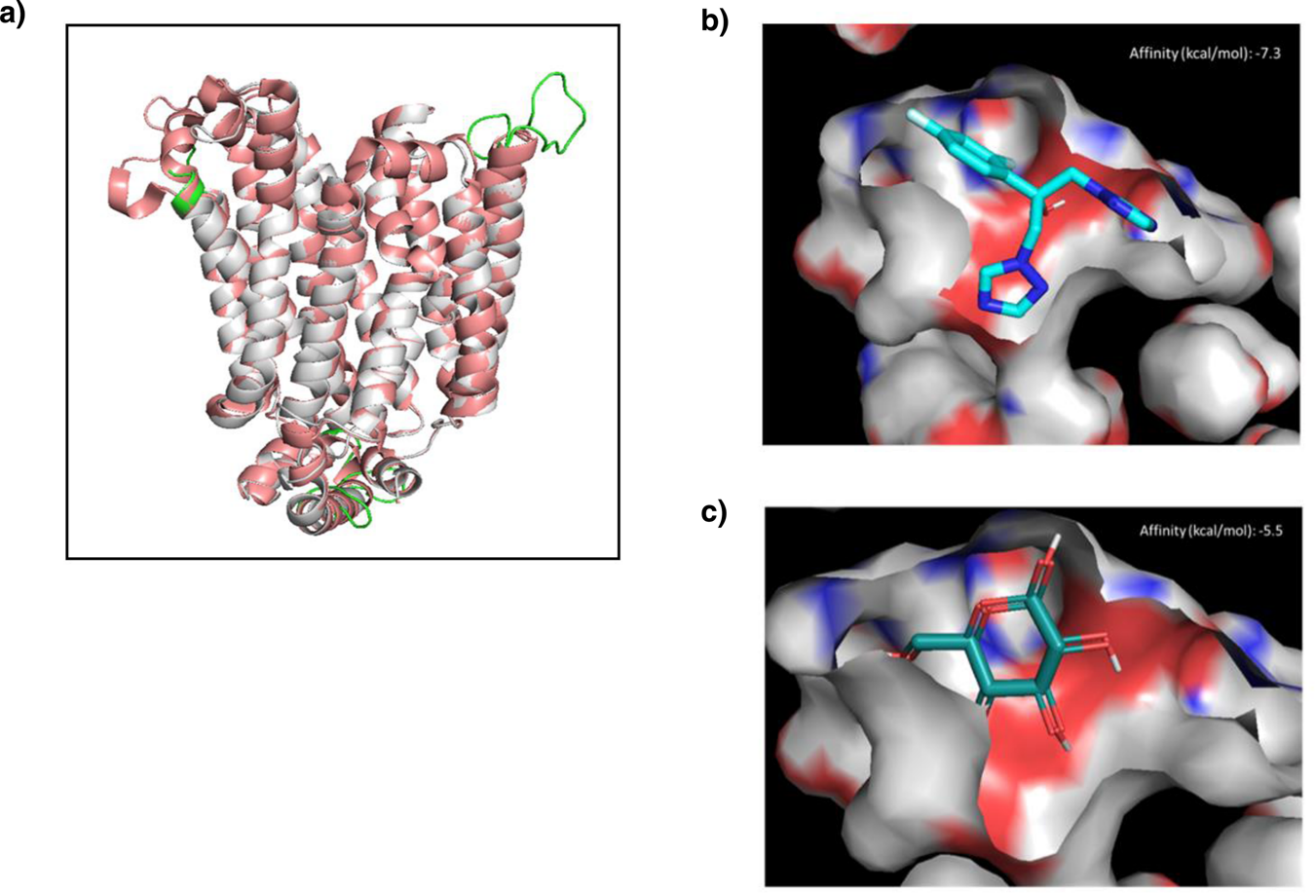
Fluconazole displays a strong predicted affinity for the glucose binding site of CgHxt4/6/7. **a** Overlap of the best DOPE score model and the template *E. coli* XylE (PDB ID 4GBZ) (pink). Loop regions that differ from the template are represented in green. **b** Docking of fluconazole on the modelled CgHxt4/6/7 structure, best pose. **c** Docking of D-glucose on the modelled CgHxt4/6/7 structure, best pose. Figures were prepared using PyMOL2.5.

To strengthen our current hypothesis that CgHxt4/6/7 may act as an azole importer, the affinity of this hexose transporter to azoles was evaluated in silico. Since there is no available structure for CgHxt4/6/7, the CgHxt4/6/7 structure was modelled based on the 3D structure of the homologous *E. coli* transporter XylE (PDB ID 4GBZ) (Supplementary Fig. 4a). This was possible due to the sequence identity between both proteins, which is of 30% for all residues and 50% for the residues exposed to the transporting channel (Supplementary Fig. 4c). The crystal structure of *E. coli* XylE was solved with a resolution of 2.89 Å and features a D-glucose bound to its internal cavity (Supplementary Fig. 4b), providing information about the positioning of the ligands to be transported. Most of the structural differences between our model and the *E. coli* XylE crystal structure are found in the loops that connect the transmembrane helices (Fig. 6a). These loops are unstructured and exposed to the solvent regions that are far from the substrate binding regions, therefore not accountable for the quality of the model at the transmembrane region (Fig. 6a). As observed for the *E. coli* XylE (PDB ID 4GBZ) protein, our model is in an outward-facing, partially occluded conformation with a D-glucose molecule captured in the transmembrane channel. The ligand is trapped within the centre of the transmembrane domain, completely occluded from the intracellular side, yet solvent-accessible from the extracellular side through a channel that is too narrow (5.2 Å diameter) to allow the escape of the ligand^31^.

Using the modelled structure of CgHxt4/6/7, its affinity to fluconazole was predicted through in silico docking, using AutoDock Vina, and compared to that of glucose, the natural substrate of this transporter. This study revealed that CgHxt4/6/7 is predicted to have strong affinity to glucose (−5.5 kcal/mol), as expected, and an even stronger predicted affinity to fluconazole (−7.3 kcal/mol) (Fig. 6b, c). Similar binding affinities to glucose and fluconazole were observed for the *E. coli* XylE transporter (Supplementary Fig. 5a, b). Although occupying the same region in the internal cavity, fluconazole and D-glucose revealed distinct patterns of interactions with the protein in our docking studies. D-glucose participates in more hydrogen bonds than fluconazole, whereas desolvation effects seem to play a major role in the binding affinity of the latter. Indeed, fluconazole shows a higher shape complementarity to the binding pocket compared to D-glucose (Fig. 6b, c). Taken together, our results suggest that Fluconazole is predicted to be a binder of CgHxt4/6/7. Despite these results, posaconazole docking failed to bind to the transporter, what would be expected given the size difference between the fluconazole and posaconazole. However, it is important to consider that this modelling was made from a partially occluded conformation, and, as such we can hypothesize that posaconazole may be accommodated and transported in an open conformation. In order to test this possibility, we decided to make a second modelling from the structure of *Homo sapiens* glucose transporter (PDB ID 4ZWC)^32^ that was obtained in an outward-open conformation (Supplementary Fig. 6). Indeed, docking studies showed that the open configuration allows binding of both posaconazole and fluconazole. Interestingly, both drugs bind in the same region in the outward-open structure (Supplementary Fig. 7). It is important to mention that posaconazole is a large molecule with a higher number of possible rotations, which makes it difficult to predict the precise mode of binding, beyond the base structure common to fluconazole, through molecular docking.

## Discussion

In this study, two hexose transporters in *C. glabrata* were identified and characterized. More importantly, a possible role for hexose transporters as drug carriers is proposed in *C. glabrata*, shedding light into an important feature of azole drug mode of action: its uptake into fungal cells, possibly through hexose transporters. An in vitro directed evolution experiment was used to drive susceptible clinical isolates towards multi azole resistance, leading to the functional characterization of the CgHxt4/6/7 hexose transporter as a determinant of azole susceptibility in *C. glabrata*. The in vitro evolved resistant isolates 040_Psc, 044_Psc and OL152_Psc acquired multi azole resistance through previously unknown mechanisms. No *CgPDR1* GOF mutations were found in these evolved strains, the main mechanism of acquired azole resistance in *C. glabrata* clinical isolates^4, 20^. Moreover, according to the general notion that alterations on the drug target are not a frequent mechanism of acquired azole resistance in *C. glabrata* clinical isolates^5, 9, 21^, we confirmed that no mutations arose in the *CgERG11* gene during posaconazole exposure. Although there were no common nsSNPs among the three evolved resistant strains, *CgHXT4/6/7* gene was found to be mutated in all three evolved resistant strains (Supplementary Fig. 2b). Our results show that, although *CgPDR1* GOF mutations are a frequent event leading to acquisition of azole resistance, they are not the only mechanism. Whether the posaconazole-driven evolution, when compared to evolution driven by other azole drugs, or the genetic background of the used strains may underlie the results obtained in this study remains unclear and is certainly worthy of further scrutiny.

CgHxt4/6/7 was, so far, an uncharacterized putative hexose transporter in *C. glabrata*. Based on knowledge gathered for *S. cerevisiae* homologous transporters, CgHxt4/6/7 belongs to the big family of *HXT* genes, specifically to the high-affinity glucose transporter cluster^33^. With 96% amino acid sequence identity to CgHxt4/6/7, CgHxt6/7 was also characterized. According to their predicted function, CgHxt4/6/7 and CgHxt6/7 were found to be localized to the cell plasma membrane (Fig. 1a), and to be able to import hexoses (Fig. 1b).

Several studies have covered a panoply of drug resistance mechanisms that depend on drug efflux pumps belonging to the ABC and MFS. Nonetheless, despite some efforts^25, 34^, the study of drug uptake mechanisms has been, to some extent, overlooked in pathogenic fungi^24^. Besides being an important mechanism of drug effectiveness acknowledged in human parasites^24^, decreased accumulation of drugs have been associated with azole resistance in *Candida* clinical isolates^25^. Nonetheless, the players underlying drug uptake have remained elusive for at least 20 years. *HXT* genes belong to the MFS class of membrane proteins and were associated with the multidrug resistance phenomenon in *S. cerevisiae* in 1997, being implicated in cycloheximide, sulfomethuron methyl and 4-NQO (4-nitroquinoline-*N*-oxide) susceptibility^35^. In fact, CgHxt4/6/7 and CgHxt6/7 *S. cerevisiae* orthologues (ScHxt6 and ScHxt7, respectively) were previously shown to be major mediators of arsenic import in yeast^36^. Our study shows that at least some of *S. cerevisiae HXT* transporters also play a relevant role in yeast azole susceptibility, probably by being hijacked by these antifungals serving as channels for drug entry into fungal cells (Fig. 3). Consistently, CgHxt4/6/7 or CgHxt6/7 expression was found to confer azole susceptibility to *C. glabrata* cells (Fig. 2a). The accumulation of radiolabelled fluconazole was shown to be higher in wild-type *C. glabrata* cells when compared to cells devoid of *CgHXT4/6/7* (Fig. 2b), suggesting that fluconazole can enter *C. glabrata* cells through facilitated diffusion, mediated by this transporter. Furthermore, in silico molecular docking results corroborate this hypothesis, as they predict that CgHxt4/6/7 has affinity to its natural substrate, glucose, but also affinity to fluconazole and posaconazole. Interestingly, the CgHxt4/6/7 related phenotypes were observed in the *C. glabrata* lab strain KUE100, but also in the 040 clinical isolate (Fig. 4a, b), suggesting that the role of this transporter is not strain dependent. Additionally, the Val121Ile mutation that arose in the 040 clinical isolate upon prolonged exposure to posaconazole was found to render this transporter inactive in terms of azole susceptibility, suggesting that similar point mutations may be used as clinically relevant mechanisms of evolution towards azole resistance (Fig. 4c).

In wild-type *C. glabrata* cells, the prime mechanism of azole drug resistance involves CgPdr1^3^. Once this transcription factor acquires GOF mutations, induced by drug exposure, natural selection favours cells harbouring these mutations, ultimately leading to the disappearance of other subpopulations and to the development of fully azole-resistant cells^20, 28^. Therefore, *CgPDR1* disruption in *C. glabrata* cells restores azole susceptibility^4^. Since there were no GOF mutations in *CgPDR1* in the evolved resistant strains 040_Psc, 044_Psc and OL152_Psc, we assessed the impact of the proposed resistance mechanism, involving the reduced uptake of fluconazole, in a strain devoid of *CgPDR1*. While in wild-type cells the single deletion of CgHxt4/6/7 or CgHxt6/7 results only in a moderate increase in azole resistance, in cells devoid of CgPdr1 the single deletion of either *HXT* gene fully reverses the strong azole susceptibility phenotype imposed by the absence of CgPdr1 (Fig. 5a, b). This further supports the concept that azoles may enter *C. glabrata* cells through Hxt proteins and that the impairment of this pathway constitutes an important mechanism of resistance, that, when triggered, renders alternative azole resistance pathways, based on drug extrusion, almost irrelevant.

Overall, we show that hexose transporters affect azole accumulation in *C. glabrata* and play a role in the acquisition of resistance by clinical isolates as an alternative to the main mechanism of acquired azole resistance involving *CgPDR1*. Despite the extensive knowledge on the mechanisms of action/resistance to azoles, little was known about how these drugs enter fungal cells to exert their antifungal action. Although indirect effects of *HXT* transporters in azole susceptibility, eventually affecting energy availability or the expression of drug efflux pumps, cannot be fully discarded, our results show that CgHxt4/6/7 and CgHxt6/7 transporters not only play a role in the uptake of glucose, but also appear to mediate azole uptake into *C. glabrata* cells. Being two in a family of 11 *HXT* transporters in *C. glabrata*, it is likely that their role in azole uptake is just the tip of the iceberg. It will be important to assess how far other homologous hexose transporters may contribute to azole uptake and whether mutations acquired in additional hexose transporter encoding genes may contribute to azole resistance in fungal pathogens, in general.

## Methods

### Strains and growth media

The reference strain CBS138 and three *Candida glabrata* clinical isolates (named OL152, 040 and 044) obtained from patients admitted to Centro Hospitalar de S. João (CHSJ) in Porto, Portugal were used in this study^27^. Isolates 040 and 044 were recovered from blood cultures, while isolate OL152 was recovered from urine. These isolates were characterized by Vitek YBC identification cards (BioMérieux, Paris, France) and mass-spectrometry (Maldi TOF). Until testing, the strains were stored in Brain–Heart broth (Difco) with 5% glycerol at −70 °C. For each experiment, the strains were subcultured twice on Sabouraud agar (Difco) at 35 °C for 48 h.

*Candida glabrata* parental strain KUE100^37^ and derived KUE100_Δ*cgpdr1*^38^ were kindly provided by Hiroji Chibana, Medical Mycology Research Center at Chiba University, Japan. KUE100_Δ*cghxt4/6/7*, KUE100_Δ*cghxt6/7*, KUE100_Δ*cghxt4/6/7*Δ*cghxt6/7*, KUE100_Δ*cgpdr1*Δ*cghxt4/6/7*, KUE100_Δ*cgpdr1*Δ*cghxt6/7*, and KUE100::URA-, were all constructed in this study. Cells were batch-cultured at 30 °C, with orbital agitation (250 rpm) in Basal Medium (BM) or Yeast Extract-Peptone-Dextrose (YPD) medium. BM has the following composition (per litre): 1.7 g yeast nitrogen base without amino acids or NH_4_^+^ (Difco), 20 g glucose (Merck) and 2.65 g (NH_4_)_2_SO_4_ (Merck). YPD has the following composition (per liter): 20 g glucose (Merck), 20 g Peptone (Merck) and 10 g Yeast extract (Merck).

*Saccharomyces cerevisiae* parental strain BY4741 (*MATa*, *ura3∆0*, *leu2∆0*, *his3∆1*, *met15∆0*) was obtained from the Euroscarf collection. Wild-type yeast cells harbouring pGREG576 derived plasmids were batch-cultured at 30 °C, with orbital agitation (250 rpm) in BM supplemented with 20 mg L^−1^ methionine, 20 mg L^−1^ histidine and 60 mg L^−1^ leucine (all from Sigma).

*S. cerevisiae* parental strain CEN.PK2-1C^39^ and derived hexose transporter-null (*Schxt*^*0*^) strain EBY.VW4000^29^ were kindly provided by Eckhard Boles, Institute of Molecular Biological Sciences, Goethe University, Frankfurt, Germany. Cells were batch-cultured at 30 °C, with orbital agitation (250 rpm) in Yeast Extract-Peptone-Maltose (YPM) medium containing (per liter): 20 g maltose (Merck), 20 g Peptone (Merck) and 10 g Yeast extract (Merck). CEN.PK2-1C and EBY.VW4000 cells harbouring pGREG576 derived plasmids were grown overnight in BM-Maltose medium containing (per liter): 1.7 g yeast nitrogen base without amino acids or NH_4_^+^ (Difco), 20 g maltose (Merck) and 2.65 g (NH_4_)_2_SO_4_ (Merck), supplemented with 20 mg L^−1^ tryptophan, 20 mg L^−1^ histidine and 60 mg L^−1^ leucine (all from Sigma).

Solid media contained, besides the above indicated ingredients, 20 g L^−1^ agar (Iberagar).

### Gene expression analysis

The transcript level of the *CgERG11* gene in the initial azole susceptible clinical isolates was compared to the evolved resistant strains using quantitative real-time PCR (qRT-PCR). Total-RNA samples were obtained from cell suspensions harvested upon reaching an OD_600nm_ = 0.8 ± 0.08 in rich YPD medium. cDNA for real-time reverse transcription-PCR was synthesized from total-RNA samples by using the MultiScribeTM reverse transcriptase kit (Applied Biosystems) and the 7500 RT-PCR thermal cycler block (Applied Biosystems) according to the manufacturer’s instructions. The quantity of cDNA for subsequent reactions was kept at ca. 10 ng. The subsequent RT-PCR step was carried out using SYBR green reagents. Primers for the amplification of the *CgERG11* and *CgACT1* genes were designed using Primer Express software (Applied Biosystems) and are summarized in Supplementary Table 3. The RT-PCR was carried out using a thermal cycler block (7500 real-time PCR system, Applied Biosystems). Default parameters established by the manufacturer were used, and fluorescence was detected by the instrument and recorded in an amplification plot (7500 System SDS software, Applied Biosystems). The *CgACT1* mRNA level was used as an internal control. The relative values obtained for the initial isolates exhibiting the lower gene expression level were set as 1 and the remaining values are presented relative to that control. To avoid false-positive signals, the absence of nonspecific amplification with the chosen primers was confirmed by the generation of a dissociation curve for each pair of primers.

### Plasmids

For *CgHXT4*/6/7 or *CgHXT*6/7 gene cloning, the plasmid pGREG576 was obtained from the Drag&Drop collection^40^. For *CgHXT4*/6/7 or *CgHXT*6/7 gene disruption, the plasmid pYC44 (Addgene plasmid #63903) was used; for expression of the flippase enzyme, the plasmid pLS10 was used. For *CgURA3* gene disruption, the plasmid pV1382^41^ was obtained from Addgene (Addgene plasmid #111436).

### Disruption of *CgHXT4/6/7* (ORF *CAGL0A02233g*) or *CgHXT6/7* (ORF *CAGL0A02211g*)

The wild-type strain KUE100, derived KUE100_*Δcgpdr1* mutant, and 040 clinical isolate were transformed by electroporation with the deletion cassette (a nourseothricin cassette flanked by FRT sites and a ~500 bp region flanking the targeted gene). The deletion cassette was constructed by Gibson Assembly using the pYC44 plasmid, previously cut with XhoI and Not1, ~500 bp of the *CgHXT4/6/7* promoter region and ~500 bp of the *CgHXT4/6/7* terminator region, or ~500 bp of the *CgHXT6/7* promoter region and ~500 bp of the *CgHXT6/7* terminator region. Cells were plated on YPD agar medium supplemented with 200 mg L^−1^ nourseothricin. Transformants were checked for insertion of the deletion cassette by PCR using the control primers. Correct strains were subsequently transformed with plasmid pLS10 to induce expression of the flippase enzyme (300 mg L^−1^ hygromycin selection). Removal of the nourseothricin cassettes of the transformants was checked by PCR. The pLS10 plasmid was lost by growth on non-selective YPD medium and checked by replating on YPD supplemented with 300 mg L^−1^ hygromycin. All the primers used for the construction of the mutant strains are depicted in Supplementary Table 3.

### Cloning of the *CgHXT4/6/7* (ORF *CAGL0A02233g*) or *CgHXT6/7* (ORF *CAGL0A02211g*)

The pGREG576 plasmid from the Drag&Drop collection was used to clone and express *CgHXT4/6/7* or *CgHXT6/7* genes, as described before^14–17, 19^. pGREG576 contains a galactose inducible promoter (*GAL1*), the yeast selectable marker *URA3* and the *GFP* gene, encoding a Green Fluorescent Protein (GFPS65T), which allows monitoring of the expression and subcellular localization of the cloned fusion protein. *CgHXT4/6/7* and *CgHXT6/7* DNA was generated by PCR, using genomic DNA extracted from the sequenced CBS138 *C. glabrata* strain. The designed primers contain, besides a region with homology to the first ~25 and last ~25 nucleotides of the *CgHXT4/6/7* or *CgHXT6/7* coding region, nucleotide sequences with homology to the cloning site flanking regions of the pGREG576 vector. The amplified fragments were co-transformed into the parental *S. cerevisiae* strain BY4741 with the pGREG576 vector, previously cut with the restriction enzyme SalI, to obtain the pGREG576_*CgHXT4/6/7* or pGREG576_*CgHXT6/7* plasmids. Since the *GAL1* promoter needs galactose for induction in *S. cerevisiae*, and only allows a slight expression of downstream genes in *C. glabrata*, new constructs were obtained. The *GAL1* promoter was replaced by the pPDC1 *C. glabrata* constitutive promoter^42^, generating the pGREG576_PDC1_*CgHXT4/6/7* or pGREG576_PDC1_*CgHXT6/7* plasmids, or by the TEF *S. cerevisiae* constitutive promoter^43^, generating the pGREG576_TEF_*CgHXT4/6/7* or pGREG576_TEF_*CgHXT6/7* plasmids. The *PDC1* promoter DNA was generated by PCR, using genomic DNA extracted from the sequenced CBS138 *C. glabrata* strain. The TEF promoter DNA was generated by PCR, using genomic DNA extracted from the sequenced BY4741 *S. cerevisiae* strain. The amplified fragments were co-transformed into the parental strain BY4741 with the pGREG576_*CgHXT4/6/7* or pGREG576_*CgHXT6/7* plasmid, previously cut with SacI and NotI restriction enzymes to remove the *GAL1* promoter. The recombinant plasmids were obtained through homologous recombination in *S. cerevisiae* and verified by DNA sequencing. All primers used are present in Supplementary Table 3.

### Site-directed mutagenesis

The *CgHXT4/6/7* gene sequence in the recombinant pGREG576_PDC1_*CgHXT4/6/7* plasmid was mutated by site-directed mutagenesis. The designed primers contain the mutation occurred in the 040_Psc resistant isolate during posaconazole exposure, resulting in the production of the mutated sequence by PCR amplification to obtain the pGREG576_PDC1_mut_*CgHXT4/6/7* plasmid. The original template was then degraded by DpnI digestion. All primers used are present in Supplementary Table 3.

### Disruption of the *CgURA3* (ORF *CAGL0I03080g*)

The disruption of the *C. glabrata URA3* gene, encoded by ORF *CAGL0I03080g*, was carried out in the KUE100 parental strain as described previously^44^, using the CRISPR-Cas9 system from Vyas et al.^41^ Briefly, a *CgURA3* gRNA sequence selected from the resources made available by Vyas et al.^41^ was cloned in the pV1382 plasmid, previously linearized with the restriction enzyme BsmBI. The *CgURA3* gRNA was obtained by oligonucleotide annealing and the product ligated into the previously linearized pV1382 plasmid to obtain the pV1382_*CgURA3* vector. The construct was verified by DNA sequencing. The plasmid was transformed into *C. glabrata* cells which were then directly plated on 5-Fluoroorotic acid (5-FOA) to select for URA- cells. Sequential passages in nonselective medium (YPD) were performed to avoid detrimental effects of further Cas9 expression and *CgURA3* loss of function was further confirmed by the inability to grow in medium without uracil. The introduction of pGREG576 derived plasmids in the edited strains was able to rescue the growth impairment in the absence of uracil. Sequencing of the *CgURA3* gene from the selected candidates revealed the existence of frameshifts within the ORF, thus resulting in premature stop codons as it is expected from Non-Homologous End Joining (NHEJ) correction of double-strand breaks. All primers used are present in Supplementary Table 3.

### In vitro induction of antifungal resistance in *C. glabrata* clinical isolates and CBS138 strain and assessment of its stability

Standard powders of fluconazole (Pfizer, Groton, CT), voriconazole (Pfizer, New York, NY), posaconazole (Schering-Plough, Kenilworth, NJ) and clotrimazole (Sigma, St. Louis, MO) were obtained from the respective manufacturers. A stock solution of fluconazole was prepared in distilled water, while voriconazole, posaconazole and clotrimazole were prepared with dimethyl sulfoxide (Sigma, St. Louis, MO). Antifungal drugs were diluted afterwards with Roswell Park Memorial Institute 1640 medium (RPMI 1640; Sigma, St. Louis, MO) buffered to pH 7.0 with 0.165 M morpholine propanesulfonic acid buffer (MOPS; Sigma) and stored at −70 °C until further use. The culture medium used for resistance induction assays was RPMI 1640 with 0.165 M MOPS, pH 7.0. A single, randomly selected, colony from each *C. glabrata* strain (040, 044, OL152 and CBS138) was incubated in 10 mL of RPMI 1640 overnight in a rotating drum at 150 rpm and 35 °C. An aliquot of this culture, containing 10^6^ yeast cells, was transferred to different vials, each containing 10 mL of culture medium with or without posaconazole and incubated overnight as described above. The following day, aliquots from each culture containing 10^6^ yeast cells were again transferred into fresh medium containing the same antifungal and re-incubated as described. Each day, for the 30 days of the assay, a 1 mL aliquot from each subculture was mixed with 0.5 mL of 50% glycerol and frozen at −70 °C for later testing. Incubation took place for 30 days with constant concentrations of posaconazole (1 mg L^−1^), corresponding to therapeutic plasma levels obtained during antifungal treatment^45^. To assess resistance stability, the evolved resistant isolates obtained were subcultured daily in the absence of the drug for 30 days. A single colony from each isolate was incubated in 10 mL drug-free RPMI 1640 at 35 °C and 150 rpm. In the following day, aliquots were transferred into fresh medium. At each subculture, a 1 mL aliquot of the suspension was mixed with 0.5 mL of 50% glycerol, and frozen at −70 °C, for further testing.

### Genome sequencing and data analysis

The draft genome sequences of the three azole susceptible *C. glabrata* clinical isolates used in this study, 040, 044 and OL152, had been characterized previously, as described in Pais et al.^46^. The genome sequences of the selected in vitro evolved resistant strains 040_Psc, 044_Psc and OL152_Psc derived from 040, 044 and OL152 clinical isolates, respectively, were obtained and characterized in this study. Raw sequencing data and genome assemblies can be found accessing BioProject no. PRJNA525402 (initial isolates) and BioProject no. PRJNA694431 (evolved isolates).

Genome sequencing was performed by Illumina HiSeqX 150-bp, paired-end sequencing. Library preparation (Nextera XT) and sequencing were carried out by Admera Health, LLC. Each resistant isolate was confirmed to be the same as their correspondent initial isolate by Multi Locus Sequence Typing (MLST) using six *loci* (*FKS*, *LEU2*, *NMT1*, *TRP1*, *UGP1*, *URA3*)^47^ and each sequence typing (ST) was identified according to the Sequence Typing website^48^(https://pubmlst.org/organisms/candida-glabrata). Genome assembly and comparison with the *C. glabrata* reference genome was carried out as described previously^46^. Briefly, raw sequencing reads were trimmed, and duplicates removed to only keep high-quality reads. Reads were mapped against the *C. glabrata* reference genome yielding >97% of aligned reads for each isolate. Genomes were assembled using SPAdes^49^ and scaffolds <500 bp discarded to attain final draft genomes. Each assembly was aligned against the reference genome and found to align >97.6% in each case. The high sequencing depth was leveraged to performed Single Nucleotide Polymorphism (SNP) identification with increased sensitivity and sensibility across the genome. Briefly, reads were aligned against the reference genome and variant identification was performed using GATK^50^. Low-quality variants were subsequently filtered out with BCFtools^51^ as described previously^46^. For subsequent analysis, only variants occurring within gene coding sequences and resulting in missense or non-sense mutations in the encoded protein were considered.

### Antifungal susceptibility assays

#### Clinical isolates

The Minimal Inhibitory Concentration (MIC_50_) of voriconazole, fluconazole, clotrimazole and posaconazole antifungal drugs was determined according to the M27-A3 protocol of the Clinical Laboratory Standards Institute^52^, for all the *C. glabrata* isolates under study. The MICs were determined after 24 h. Interpretative criteria for fluconazole were those of the CLSI document M60^53^: susceptible-dose dependent (S-DD)-MIC_50_≤32 mg L^−1^ and resistance (R)-MIC_50_≥64 mg L^−1^. Although susceptibility breakpoints have not yet been established for voriconazole, posaconazole or clotrimazole for *C. glabrata*, strains inhibited by ≤2m L^−1^, ≤1m L^−1^ and ≤2m L^−1^, respectively, were considered to be susceptible. Every 5 days of incubation, with or without antifungal, MIC_50_ values were redetermined for the four antifungals tested. The antifungal concentrations ranged from 0.03125 to 16 mg L^−1^ for posaconazole and voriconazole, and 0.125-64 mg L^−1^ for fluconazole and clotrimazole. *C. glabrata* reference strain CBS138 was used in each testing assay, as recommended.

#### Lab strains

*C. glabrata* cells susceptibility to toxic concentrations of the selected azoles was evaluated by spot assays. Cell suspensions used to inoculate agar plates were prepared with mid-exponential cells grown in YPD, YPM or BM until an OD_600nm_ = 0.5 ± 0.05, and then diluted in sterile water to obtain suspensions with an OD_600nm_ = 0.05 ± 0.005. These cell suspensions and subsequent 1:5 and 1:25 dilutions were applied as 4 µL spots onto the surface of appropriate solid media supplemented with adequate concentrations of chemical stressors. The drugs tested included the following compounds, used in the specified concentrations: fluconazole 10, 100 or 150 mg L^−1^, posaconazole 2 or 4 mg L^−1^ and Itraconazole 1 mg L^−1^ (all from Sigma).

Yeast cells used for susceptibility assays in liquid medium were grown overnight in YPD broth medium at 30 °C, 250 rpm. New cultures were made in fresh YPD medium, supplemented or not with 4 mg L^−1^ of posaconazole, with an initial OD_600nm_ = 0.01 ± 0.001. Growth, taking place in Erlenmeyer flasks, at 30 °C, 250 rpm, was followed by measuring the optical density of the cell suspension at 600 nm. Drug concentrations used for spot and liquid susceptibility assays using YPD medium were higher than the standard range used for MIC determination since YPD is a richer medium, comparing to RPMI, and the initial cell concentration used was higher.

Posaconazole and fluconazole MIC_50_ values for each strain were determined in accordance with the CLSI guidelines by using 96-well plates containing RPMI 1640 2% glucose medium supplemented with the appropriate drug concentration. The antifungal concentrations ranged from 0.015625 to 8 mg L^−1^ for posaconazole and 0.125–64 mg L^−1^ for fluconazole.

### CgHxt4/6/7 or CgHxt6/7 subcellular localization assessment

The subcellular localization of the CgHxt4/6/7 or CgHxt6/7 protein was determined based on the observation of KUE100::URA- *C. glabrata* cells and BY4741 *S. cerevisiae* cells transformed with pGREG576_PDC1_*HXT4/6/7* or pGREG576_PDC1_*HXT6/7*, and pGREG576_TEF_*CgHXT4/6/7* or pGREG576_TEF_*CgHXT6/7* plasmids, respectively. These cells express either the CgHxt4/6/7_GFP or the CgHxt6/7_GFP fusion protein, whose localization may be determined using fluorescence microscopy. *C. glabrata* or *S. cerevisiae* cells were grown in BM without uracil, for plasmid maintenance, until mid-exponential phase, OD_600nm_ = 0.5 ± 0.05. The distribution of fusion protein was assessed by fluorescence microscopy in a Zeiss Axioplan microscope (Carl Zeiss MicroImaging), using excitation and emission wavelength of 395 and 509 nm, respectively. Fluorescence images were captured using a cooled Zeiss Axiocam 503 colour (Carl Zeiss Microscopy).

### Hexose utilization assays

The ability of CgHxt4/6/7 and CgHxt6/7 to import glucose, mannose, fructose or galactose into fungal cells was accessed in *S. cerevisiae* by using the hexose transporter-null EBY.VW4000 (*Schxt*^*0*^) strain, which grows normally on maltose, and barely on galactose as carbon sources. EBY.VW4000 cells harbouring pGREG576 empty vector or the pGREG576_TEF_*CgHXT4/6/7* or pGREG576_TEF_*CgHXT4/6/7* plasmids were grown overnight at 30 °C, with orbital agitation (250 rpm) in BM-Maltose, without uracil. Cell suspensions used to inoculate agar plates were mid-exponential cells grown until an OD_600nm_ = 0.5 ± 0.05, and then suspended in sterile water to an OD_600nm_ = 0.05 ± 0.005. These cell suspensions and subsequent 1:5 and 1:25 dilutions were applied as 4 µL spots onto the surface of solid BM media plates containing the indicated proportion of carbon sources, supplemented with 20 mg L^−1^ tryptophan, 20 mg L^−1^ histidine and 60 mg L^−1^ leucine.

### [^3^H]-fluconazole and [^3^H]-clotrimazole accumulation assays

The intracellular accumulation of fluconazole or clotrimazole in the different *C. glabrata* strains was compared resorting to radiolabelled [^3^H]-fluconazole and [^3^H]-clotrimazole, respectively. The internal accumulation was determined by calculating the ratio between the value measured within yeast cells and in the external medium ([Intracellular]/[Extracellular]). The parental strains and the derived mutant strains were grown in YPD medium until mid-exponential phase and harvested by filtration. Cells were washed and resuspended in YPD medium, to obtain dense cell suspensions (OD_600nm_ = 0.5 ± 0.1, equivalent to approximately 1.57 mg(dry weight) mL^−1^). Readily, 0.1 µM of [^3^H]-fluconazole (Moravek Inc.; 1.0 mCi mL^−1^) or [^3^H]-clotrimazole (American Radiolabelled Chemicals, St. Louis, MO;1 mCi mL^−1^) and 100 mg L^−1^ of unlabelled fluconazole or 30 mg L^−1^ of unlabelled clotrimazole, respectively, were added to the cell suspensions. The intracellular accumulation of labelled fluconazole was followed for a 15 min period by filtering 200 µL of cell suspension through pre-wetted glass microfiber filters (Whatman GF/C). The filters were washed with ice-cold TM buffer [0.1 M 2-(*N*-morpholino)ethanesulfonic acid (Sigma)/41 mM Tris (Sigma)], and the radioactivity measured in a PerkinElmer Tri-Carb 2810TR liquid scintillation analyser. Extracellular ^3^H-fluconazole was estimated by radioactivity assessment of 50 µL of the supernatant. To calculate the intracellular concentration of radiolabelled fluconazole, the internal cell volume (V_i_) of the exponential cells, grown in the absence of drug and used for accumulation assays, was considered constant and equal to 2.5 µL mg (dry weight)^−1^.

### Protein modelling and molecular docking

The homology modelling of the CgHxt4/6/7 membrane protein was performed using MODELLER version 9.23^54^. The crystal structure of *E. coli* XylE (PDB ID 4GBZ)^31^ was used as a template for the outward-facing, partially occluded conformation and the crystal structure of the *H. sapiens* glucose transporter (PDB ID 4ZWC)^32^ was used for the outward-open conformation. For each modulation, twenty independent models were generated. Subsequent analysis were carried on the model with the lowest DOPE score^55^.

The docking calculations were performed using AutoDock Vina^56^, with an exhaustiveness value of 50 and in a search box centralized at the ligand of the PDB ID 4GBZ and with sizes of 16, 16 and 14 Å. For each docking calculation, the docking pose with lowest energy was used in subsequent analysis. Figures of docking results were prepared using PyMOL2.5^57^.

### Statistics and reproducibility

All experiments represent the average of three or more independent experiments. Error bars represent the standard deviation. Statistical analysis was performed using one-way analysis of variance with Tukey’s correction. Significance levels are attributed as follows: *p < 0.05, **p < 0.01, ***p < 0.001, ****p < 0.0001.

### Reporting summary

Further information on research design is available in the Nature Research Reporting Summary linked to this article.

## Supplementary information

Supplementary Information

Description of Additional Supplementary Files

Supplementary Data 1

Supplementary Data 2

Supplementary Data 3

Supplementary Data 4

Reporting Summary

## Data Availability

Raw sequencing data and genome assemblies can be found accessing NCBI BioProjects PRJNA525402 (initial isolates) and PRJNA694431 (evolved isolates). All other data are available from the corresponding author on reasonable request. Raw data underlying all graphs have been provided as Supplementary Data 4.
