# Supplementary material for: Genomic evolution towards azole resistance in Candida glabrata clinical isolates unveils the importance of CgHxt4/6/7 in azole accumulation
Source: Commun Biol. 2022 Oct 21;5:1118. doi: 10.1038/s42003-022-04087-0 (PMC9587243; doi:10.1038/s42003-022-04087-0)
Supplement: Supplementary file 1 — Supplementary Information [file 42003_2022_4087_MOESM1_ESM.pdf]

Supplementary Table 1. CBS138, OL152, 040 and 044 evolution toward multiazole resistance imposed by prolonged exposure to posaconazole. The CBS138 strain and the clinical isolates 040, 044 and OL152 were induced towards multiazole resistance by prolonged exposure to 1mg L^{-1} posaconazole. MIC_{50} levels were determined for fluconazole, voriconazole, posaconazole and clotrimazole every five days of incubation. Interpretative criteria for fluconazole were those of the CLSI document M60: susceptible-dose dependent (S-DD)-MIC_{50}≤32mg L^{-1} and resistance (R)-MIC_{50}≥64mg L^{-1}. Although susceptibility breakpoints have not yet been established for voriconazole, posaconazole or clotrimazole for *C. glabrata*, strains inhibited by ≤2mg L^{-1}, ≤1mg L^{-1} and ≤2mg L^{-1}, respectively, were considered to be susceptible. The evolved resistant strains, derived from the CBS138 strain and the 040, 044 and OL152 clinical isolates were called CBS138_Psc, 040_Psc, 044_Psc and OL152_Psc.

		**MIC_{50} / Phenotype**															
		**Fluconazole**				**Voriconazole**				**Posaconazole**				**Clotrimazole**			
**Induction with**	**Strains**	Day 0	Day 5	Day 10	Day 25	Day 0	Day 5	Day 10	Day 25	Day 0	Day 5	Day 10	Day 25	Day 0	Day 5	Day 10	Day 25
**Posaconazole**	CBS138	4/S-DD	4/S-DD	≥64/R	≥64/R	0.125/S	0.125/S	2/S	2/S	0.03/S	0.06/S	16/R	≥16/R	1/S	2/S	64/R	64/R
	OL152	8/S-DD	8/S-DD	≥64/R	≥64/R	0.5/S	0.5/S	2/S	2/S	1/S	1/S	16/R	≥16/R	4/R	4/R	8/R	8/R
	040	16/S-DD	8/S-DD	≥64/R	≥64/R	0.25/S	0.25/S	2/S	2/S	0.125/S	0.06/S	16/R	≥16/R	2/S	4/R	16/R	16/R
	044	4/S-DD	4/S-DD	4/S-DD	≥64/R	0.125/S	0.5/S	2/S	4/R	0.5/S	0.5/S	1/S	≥16/R	0.125/S	4/R	4/R	8/R

Supplementary Table 2. Genes that acquired non-synonymous SNPs during posaconazole exposure grouped by their biological function. Genes that acquired mutations during posaconazole exposure in the three evolved resistant strains were organized according to their biological function. *Candida* Genome Database (http://www.candidagenome.org/) was used to check each gene (predicted or verified) biological function.

	Biological function	Gene Name	ORF
**040_Psc**	Adhesion	*AWP1*	*CAGL0J02508g*
		AWP2	*CAGL0K00110g*
		AWP12	*CAGL0G10219g*
		AWP13	*CAGL0H10626g*
		EPA3	*CAGL0E06688g*
		EPA12	*CAGL0M00132g*
		PWP2	*CAGL0I10246g*
		PWP3	*CAGL0I10200g*
		PWP4	*CAGL0I10362g*
		PWP7	*CAGL0I10098g*
		-	*CAGL0C00968g*
		-	*CAGL0J01727g*
		-	*CAGL0J01774g*
		-	*CAGL0L00227g*
		-	*CAGL0L10092g*
	Sugar Metabolism	*HXT4/6/7*	*CAGL0A02233g*
		NRG1	*CAGL0K12078g*
	Respiration	-	*CAGL0M14091g*
	Unknown Function	-	*CAGL0K07502g*

	Biological function	Gene Name	ORF
**044_Psc**	Adhesion	*AWP2*	*CAGL0K00110g*
		AWP3	*CAGL0J11891g*
		AWP12	*CAGL0G10219g*
		EPA2	*CAGL0E06666g*
		EPA3	*CAGL0E06688g*
		EPA6	*CAGL0C00110g*
		EPA10	*CAGL0A01284g*
		EPA12	*CAGL0M00132g*
		EPA15	*CAGL0J11968g*
		PWP4	*CAGL0I10362g*
		-	*CAGL0C00253g*
		-	*CAGL0C00968g*
		-	*CAGL0C01133g*
		-	*CAGL0J01800g*
		-	*CAGL0J02530g*
		-	*CAGL0J02552g*
		-	*CAGL0L00157g*
		-	*CAGL0L00227g*
		-	*CAGL0L10092g*
		-	*CAGL0M05115g*
	Sugar Metabolism	*HXT4/6/7*	*CAGL0A02233g*
	Unknown Function	-	*CAGL0G07183g*

	Biological function	Gene Name	ORF
**OL152_Psc**	Adhesion	*AWP1*	*CAGL0J02508g*
		AWP3	*CAGL0J11891g*
		AWP12	*CAGL0G10219g*
		AWP13	*CAGL0H10626g*
		EPA2	*CAGL0E06666g*
		EPA3	*CAGL0E06688g*
		EPA6	*CAGL0C00110g*
		EPA15	*CAGL0J11968g*
		PWP1	*CAGL0I10147g*
		PWP3	*CAGL0I10200g*
		PWP7	*CAGL0I10098g*
		-	*CAGL0C01133g*
		-	*CAGL0E00231g*
		-	*CAGL0E01661g*
		-	*CAGL0G04125g*
		-	*CAGL0J01774g*
		-	*CAGL0J01800g*
		-	*CAGL0J05159g*
		-	*CAGL0L00157g*
		-	*CAGL0L00227g*
		-	*CAGL0C00968g*
	Cell-wall Metabolism	*ACK1*	*CAGL0J09702g*
	Sugar Metabolism	*HXT4/6/7*	*CAGL0A02233g*
	ER transport	*SEC16*	*CAGL0H05577g*
	Protein translation	*BUD27*	*CAGL0K07700g*
	Respiration	-	*CAGL0M14091g*
	Unknown Function	-	*CAGL0M05115g*
		-	*CAGL0G07645g*

Supplementary Table 3. List of oligonucleotides designed and used in this study for gene disruption, gene cloning and PCR-based validation.

Used to	Primer sequences
Amplify ~500 bp of the promotor region of *CgHXT4/6/7*	5'-TGGAGCTCCACCGCGGTGGCGTAGAAACAGCCTGTTCTTC -3'<br>5'- GGATCCACTAGTTCTAGAGCTTTTAATGTTAGTATTATTTTATGTCGATC -3'
Amplify ~500 bp of the promotor region of *CgHXT6/7*	5'-TGGAGCTCCACCGCGGTGGCTATCTTGTAGTTATTTAGATCTTTCC-3'<br>5'-GGATCCACTAGTTCTAGAGCTATTGTTATTTATTATTTGTAATAGTTTGAATAATC-3'
Amplify ~500 bp of the terminator region of *CgHXT4/6/7*	5'- AGAGAATAGGAACTTCGTCCATCAATTGACAGTTTAATTTGC -3'<br>5'- AGCTGGTACCGGGCCCCCCCAGTAAATTCTTAATGGTTCATTG -3'
Amplify ~500 bp of the terminator region of *CgHXT6/7*	5'-AGAGAATAGGAACTTCGTCCATCTCCTGAACCCTAATAATC-3'<br>5'-AGCTGGTACCGGGCCCCCCCTCGTCTTTTCAAATCAATTG-3'
Confirming insertion of the deletion cassette for *CgHXT4/6/7*	5'- TGGAGCTCCACCGCGGTGGCGTAGAAACAGCCTGTTCTTC -3'<br>5'-TTAATAGAATCTCTTGTACCATGGCTTGTCATCGTGCATCAAAGCGTCAGCATCG-3'
Confirming insertion of the deletion cassette for *CgHXT6/7*	5'-TGGAGCTCCACCGCGGTGGCTATCTTGTAGTTATTTAGATCTTTCC-3'<br>5'-TTAAACAAATCTCTTGTACCATGGCTTGTCATCGTGCATCATAGCGTCAGC-3'
Confirming removal of the deletion cassette for *CgHXT4/6/7*	5'- GGTTGTACGCTCATTTTACTG -3'<br>5'- TCACACTTGGTGGATAAACG -3'
Confirming removal of the deletion cassette for *CgHXT6/7*	5'-GGGAATATCATACATTCGATGAG-3'<br>5'-CTCATTCTGCGATGAGCTAC-3'
Cloning of *CgHXT4/6/7* in pGREG576	5'-GAATTCGATATCAAGCTTATCGATACCGTCGACAATGTCTGAAGAATCACAATCTGC-3'<br>5'-GCGTGACATAACTAATTACATGACTCGAGGTCGACTTAATAGAATCTCTTGTACCATGGC-3'
Cloning of *CgHXT6/7* in pGREG576	5'-GAATTCGATATCAAGCTTATCGATACCGTCGACAATGTCTGATCAAGAATCAAGATCAC-3'<br>5'-GCGTGACATAACTAATTACATGACTCGAGGTCGACTTAAACAAATCTCTTGTACCATGGC-3'
Cloning of TEF promoter in pGREG576_*CgHXT4/6/7* or pGREG576_*CgHXT6/7*	5'-TTAACCCTCACTAAAGGGAACAAAAGCTGGAGCTACACACCATAGCTTCAAAATG-3'<br>5'-<br>GAAAAGTTCTTCTCCTTTACTCATACTAGTGCGGCTTGTAATTAAAACTTAGATTAGATTGCTATG<br>-3'
Cloning of PDC1 promoter in pGREG576_*CgHXT4/6/7* or pGREG576_*CgHXT6/7*	5'-TTAACCCTCACTAAAGGGAACAAAAGCTGGAGCTAGCATTTTTATACACGTTTTAC-3'<br>5'- GAAAAGTTCTTCTCCTTTACTCATACTAGTGCGGCTGTTAATGTTTTTTGGCAATTG-3'
CgHXT4/6/7 site-directed mutagenesis	5' GTCCAAGCTAGGTGATATTTACGGTCGTAAGATC 3'<br>5' GATCTTACGACCGTAAATATCACCTAGCTTGGAC 3'
Cloning of *CgURA3* gRNA in pV1382	5'-GATCGACCGGCCAAGGTATCGTCACG-3'<br>5'-AAAACGTGACGATACCTTGGCCGGTC-3'
CgURA3 amplification and sequencing	5'-ATGTCCAGTGCCTCATATTTAC-3'<br>5'-GATGATGTTGCTAGGTATGATC-3'
qRT-PCR – *CgERG11*	5'-GCCATTGGCGCAGAGAGT-3'<br>5'- GCCATGTGATGGTGTACACAAAT-3'
qRT-PCR - *CgACT1*	5' -AGAGCCGTCTTCCCTTCCAT- 3'<br>5' -TTGACCCATACCGACCATGA- 3'

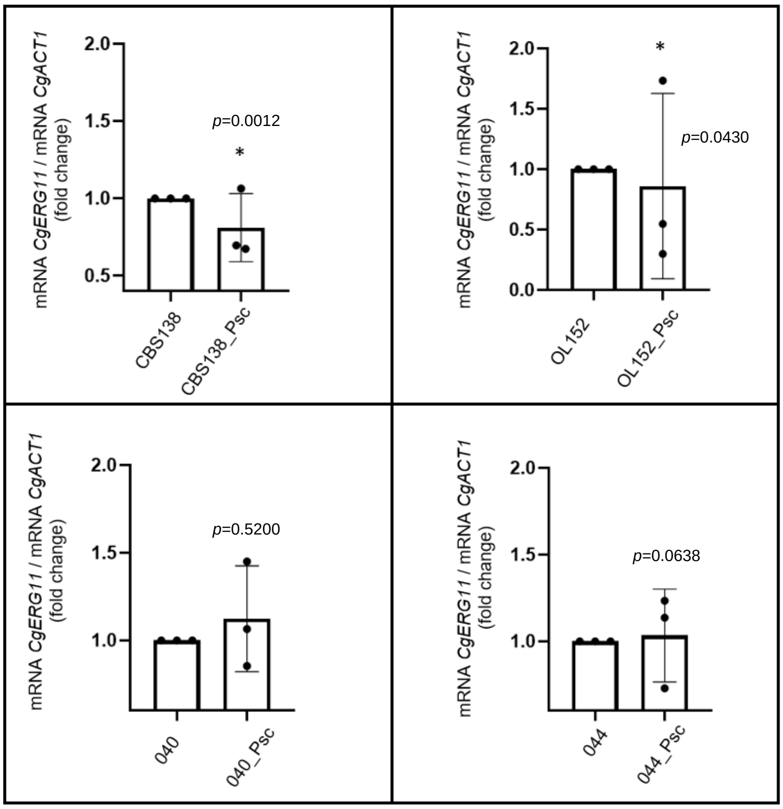

Supplementary Figure 1. *CgERG11* transcript level was not significantly altered in the evolved resistant strains. Comparison of the variation of the *CgERG11* transcript levels in the initial azole susceptible isolates 040, 044 and OL152 and CBS138 strain with the evolved resistant counterparts 040_Psc, 044_Psc, OL152_Psc and CBS138_Psc, respectively. The presented transcript levels were obtained by quantitative qRT-PCR and are *CgERG11*mRNA/*CgACT1*mRNA levels, relative to the values registered in exponentially growing cells in YPD medium. The indicated values are averages of, at least, n=3 independent experiments. Error bars represent the corresponding standard deviations. Significance levels are attributed as follows: *p < 0.05, **p < 0.01, ***p < 0.001, ****p < 0.0001.

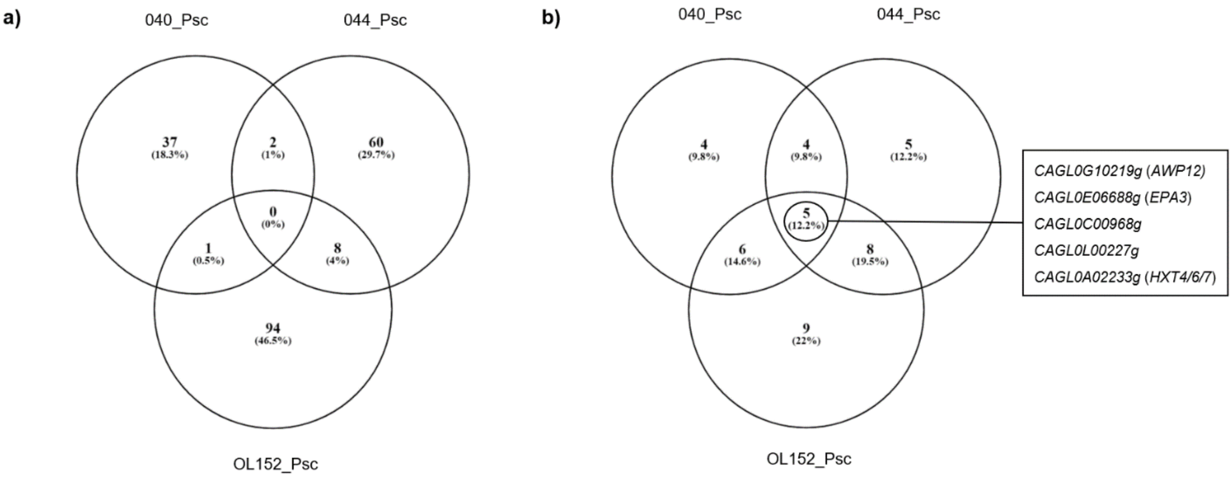

Supplementary Figure 2. Genome-wide changes occurred during posaconazole exposure. (a) Non-synonymous SNPs occurred in the evolved resistant strains 040_Psc, 044_Psc and OL152_Psc during posaconazole exposure, comparing with the parental susceptible clinical isolates 040, 044 and OL152, respectively. **(b)** Genes that acquired mutations in the evolved resistant strains 040_Psc, 044_Psc and OL152_Psc during posaconazole exposure, comparing with parental susceptible clinical isolates 040, 044 and OL152, respectively. The five commonly mutated genes among the three evolved resistant strains are indicated in the box, at the right.

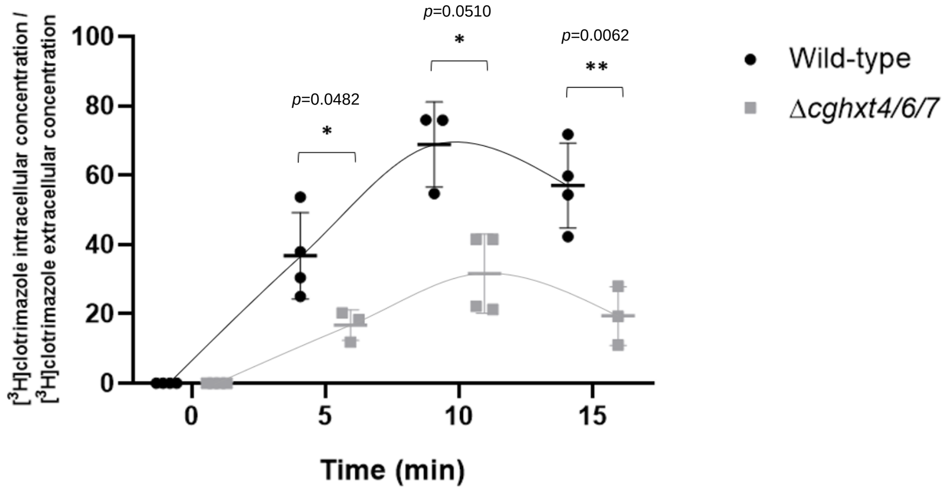

Supplementary Figure 3. CgHxt4/6/7 promotes clotrimazole accumulation in *C. glabrata* cells. Time-course accumulation ratio of [^{3}H]-Clotrimazole in non-adapted KUE100 wild-type cells (black circles) or derived KUE100_Δ*cghxt4/6/7* mutant cells (grey squares) during cultivation in liquid YPD medium in the presence of unlabeled clotrimazole. The accumulation ratio values are

averages of, at least, n=3 independent experiments. Error bars represent the corresponding standard deviations. Significance levels are attributed as follows: *$p < 0.05$, **$p < 0.01$.

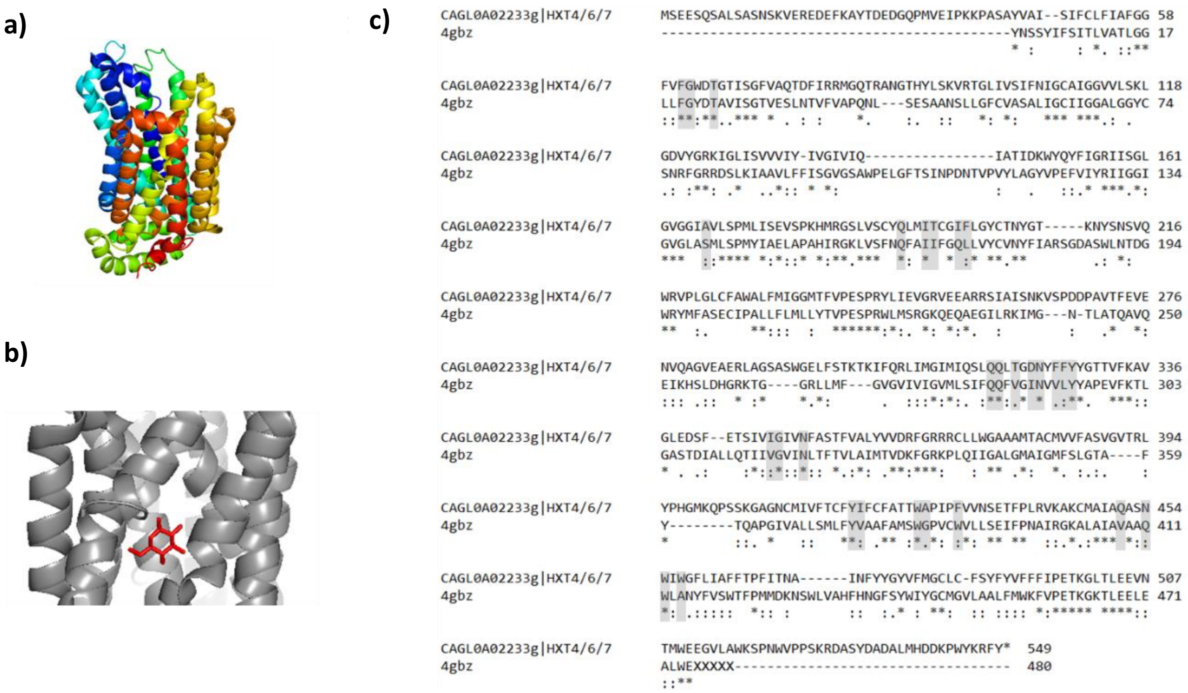

Supplementary Figure 4. CgHxt4/6/7 structure modeling based on *E. coli* XylE. (a) 3D structure of *E. coli* XylE (PDB ID 4GBZ). **(b)** Detail of 3D structure of the complex from *E. coli* XylE (PDB ID 4GBZ) with D-glucose as the ligand. **(c)** Sequence alignment of *E. coli* XylE (PDB ID 4GBZ) and *C. glabrata* CgHxt4/6/7. Residues from the center of transmembrane domain are highlighted in light gray. Figures obtained using PyMOL2.5.

a) b)

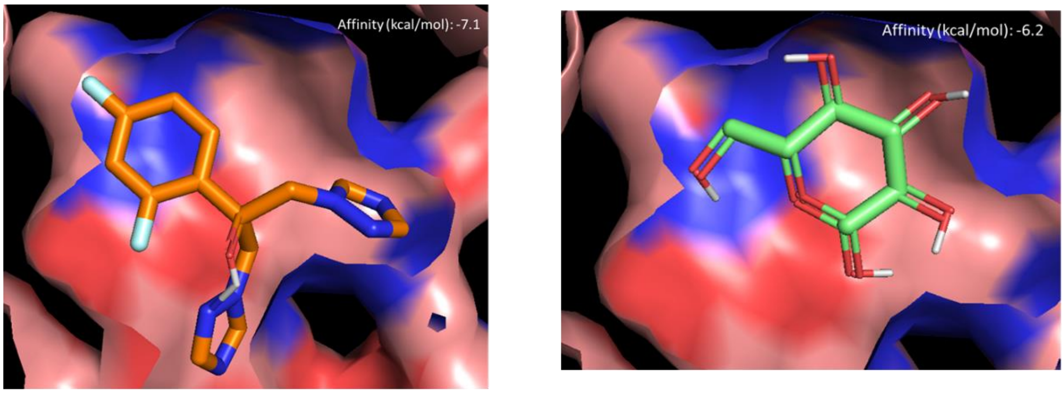

Supplementary Figure 5. Binding affinity to glucose and fluconazole on the *E. coli* XylE transporter. (a) Fluconazole docked on *E. coli* XylE (PDB ID 4GBZ). **(b)** D-glucose docked on *E. coli* XylE (PDB ID 4GBZ). Figures of docking results were prepared using PyMOL2.5.

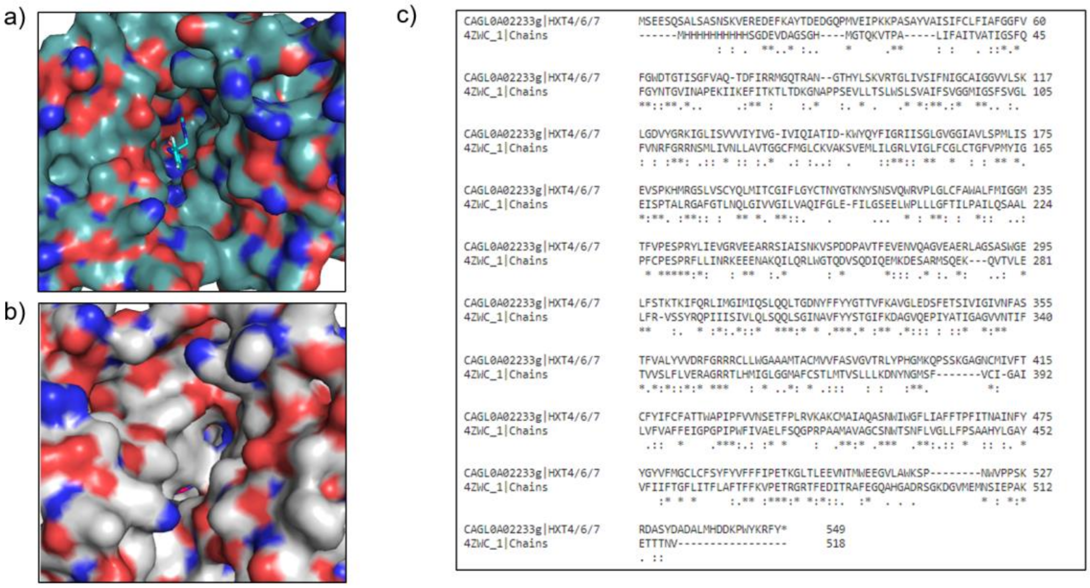

Supplementary Figure 6. CgHxt4/6/7 structure modeling based on *H. sapiens* glucose transporter (PDB ID 4ZWC). (a) and **(b)** 3D structure of the best best DOPE score model obtained of C. glabrata from homology modeling using *H. sapiens* glucose transporter (PDB ID 4ZWC) or *E. coli* XylE (PDB ID 4GBZ) as template, respectively. Figures oriented in order to visualize the transporter channel and the different conformations (a, outward-facing open; b, outward-facing partially occluded). **(c)** Sequence alignment *H. sapiens* glucose transporter (PDB ID 4ZWC) and *C. glabrata* CgHxt4/6/7. Figures obtained using PyMOL2.5.

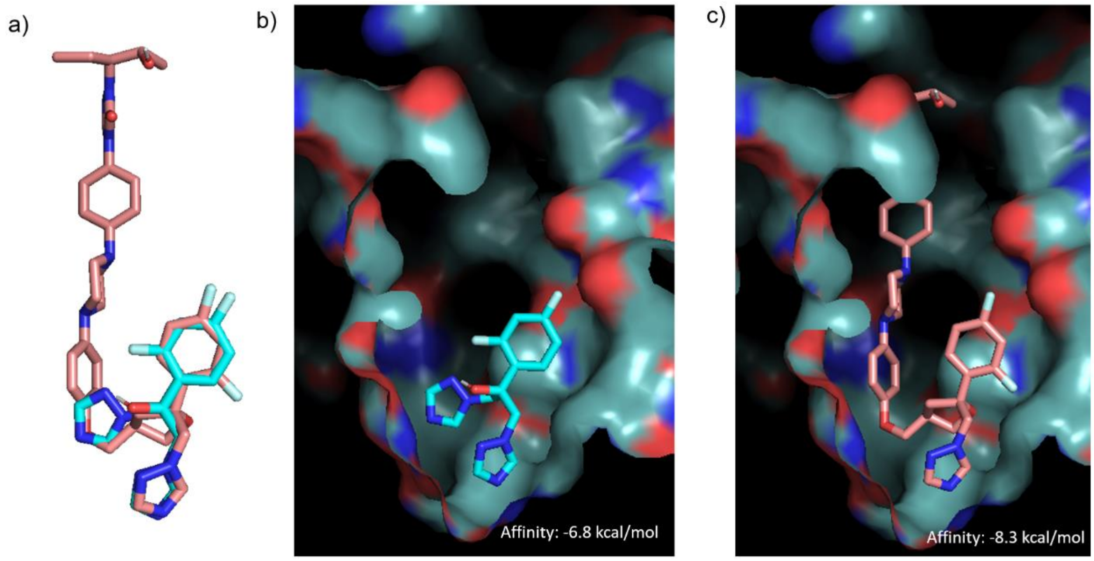

Figure 7. Open configuration allows binding of both posaconazole and fluconazole to CgHxt4/6/7. (a) Overlap of the best pose from docking of posaconazole (light pink) and Fluconazole (light blue). **(b)** Docking of fluconazole on the modeled CgHxt4/6/7 structure from *H. sapiens* glucose transporter (PDB ID 4ZWC), best pose. **(c)** Docking of D-glucose on the modeled CgHxt4/6/7 structure *H. sapiens* glucose transporter (PDB ID 4ZWC), best pose. Figures were prepared using PyMOL2.5.
