# Supplementary material for: Genomic evolution towards azole resistance in Candida glabrata clinical isolates unveils the importance of CgHxt4/6/7 in azole accumulation
Source: Commun Biol. 2022 Oct 21;5:1118. doi: 10.1038/s42003-022-04087-0 (PMC9587243; doi:10.1038/s42003-022-04087-0)
Supplement: Supplementary file 2 — Description of Additional Supplementary Files [file 42003_2022_4087_MOESM2_ESM.pdf]

Description of Additional Supplementary Files

File name: Supplementary Data 1
Description: SNPs identified in the promoters of the PDR1, CDR1 and ERG11 genes.

File name: Supplementary Data 2
Description: Non-synonimous SPNs identified in azole resistant, but not in azole susceptible strains.

File name: Supplementary Data 3
Description: SNPs identified in each strain, when compared to the reference CBS138 strain.

File name: Supplementary Data 4
Description: Source data underlying the graphs in Figs2b_4b_5b.
