# Supplementary material for: Genomic evolution towards azole resistance in Candida glabrata clinical isolates unveils the importance of CgHxt4/6/7 in azole accumulation
Source: Commun Biol. 2022 Oct 21;5:1118. doi: 10.1038/s42003-022-04087-0 (PMC9587243; doi:10.1038/s42003-022-04087-0)
Supplement: Supplementary file 7 — Reporting Summary [file 42003_2022_4087_MOESM7_ESM.pdf]

nature portfolio

Corresponding author(s): Miguel Cacho Teixeira
Acácio Gonçalves Rodrigues

Last updated by author(s): Sep 30, 2022

Reporting Summary

Nature Portfolio wishes to improve the reproducibility of the work that we publish. This form provides structure for consistency and transparency in reporting. For further information on Nature Portfolio policies, see our Editorial Policies and the Editorial Policy Checklist.

Statistics

For all statistical analyses, confirm that the following items are present in the figure legend, table legend, main text, or Methods section.

n/a	Confirmed	
☐	☒	The exact sample size (*n*) for each experimental group/condition, given as a discrete number and unit of measurement
☐	☒	A statement on whether measurements were taken from distinct samples or whether the same sample was measured repeatedly
☐	☒	The statistical test(s) used AND whether they are one- or two-sided<br>*Only common tests should be described solely by name; describe more complex techniques in the Methods section.*
☒	☐	A description of all covariates tested
☒	☐	A description of any assumptions or corrections, such as tests of normality and adjustment for multiple comparisons
☐	☒	A full description of the statistical parameters including central tendency (e.g. means) or other basic estimates (e.g. regression coefficient) AND variation (e.g. standard deviation) or associated estimates of uncertainty (e.g. confidence intervals)
☐	☒	For null hypothesis testing, the test statistic (e.g. F, t, r) with confidence intervals, effect sizes, degrees of freedom and P value noted<br>*Give P values as exact values whenever suitable.*
☒	☐	For Bayesian analysis, information on the choice of priors and Markov chain Monte Carlo settings
☒	☐	For hierarchical and complex designs, identification of the appropriate level for tests and full reporting of outcomes
☒	☐	Estimates of effect sizes (e.g. Cohen's *d*, Pearson's *r*), indicating how they were calculated

Our web collection on statistics for biologists contains articles on many of the points above.

Software and code

Policy information about availability of computer code

Data collection

Genome sequencing was performed by Illumina HiSeqX 150-bp, paired-end sequencing. Library preparation (Nextera XT) and sequencing were carried out by Admera Health, LLC.

Data analysis

Genome sequencing data analysis: Each resistant isolate was confirmed to be the same as their correspondent initial isolate by Multi Locus Sequence Typing (MLST) using six loci (FKS, LEU2, NMT1, TRP1, UGP1, URA3)47 and each sequence typing (ST) was identified according to the Sequence Typing website (https://pubmlst.org/organisms/candida-glabrata). Raw sequencing reads were trimmed, and duplicates removed to only keep high-quality reads. Reads were mapped against the C. glabrata reference genome yielding >97% of aligned reads for each isolate. Genomes were assembled using SPAdes and scaffolds <500 bp discarded to attain final draft genomes. Each assembly was aligned against the reference genome and found to align >97.6% in each case. The high sequencing depth was leveraged to performed Single Nucleotide Polymorphism (SNP) identification with increased sensitivity and sensibility across the genome. Briefly, reads were aligned against the reference genome and variant identification was performed using GATK. Low-quality variants were subsequently filtered out with BCFtools.

Protein modelling and Molecular Docking: The homology modelling of the CgHxt4/6/7 membrane protein was performed using MODELLER version 9.23. The crystal structure of E. coli XylE (PDB ID 4GBZ) was used as a template for the outward-facing, partially occluded conformation and the crystal structure of the H. sapiens glucose transporter (PDB ID 4ZWC) was used for the outward-open conformation. For each modulation, twenty independent models were generated. Subsequent analysis were carried on the model with the lowest DOPE score. The docking calculations were performed using AutoDock Vina, with an exhaustiveness value of 50 and in a search box centralized at the ligand of the PDB ID 4GBZ and with sizes of 16 Å, 16 Å and 14 Å. For each docking calculation, the docking pose with lowest energy was used in subsequent analysis. Figures of docking results were prepared using PyMOL2.5.

For manuscripts utilizing custom algorithms or software that are central to the research but not yet described in published literature, software must be made available to editors and reviewers. We strongly encourage code deposition in a community repository (e.g. GitHub). See the Nature Portfolio guidelines for submitting code & software for further information.

Data

Policy information about availability of data

All manuscripts must include a data availability statement. This statement should provide the following information, where applicable:

- Accession codes, unique identifiers, or web links for publicly available datasets
- A description of any restrictions on data availability
- For clinical datasets or third party data, please ensure that the statement adheres to our policy

Raw sequencing data and genome assemblies can be found accessing BioProject no. PRJNA525402 (initial isolates) and BioProject no. PRJNA694431 (evolved isolates). All other data are available from the corresponding author on reasonable request.

Human research participants

Policy information about studies involving human research participants and Sex and Gender in Research.

Reporting on sex and gender	Not applicable
Population characteristics	Not applicable
Recruitment	Not applicable
Ethics oversight	Not applicable

Note that full information on the approval of the study protocol must also be provided in the manuscript.

Field-specific reporting

Please select the one below that is the best fit for your research. If you are not sure, read the appropriate sections before making your selection.

☒ Life sciences ☐ Behavioural & social sciences ☐ Ecological, evolutionary & environmental sciences

For a reference copy of the document with all sections, see nature.com/documents/nr-reporting-summary-flat.pdf

Life sciences study design

All studies must disclose on these points even when the disclosure is negative.

Sample size	All experiments were conducted at least in triplicate.
Data exclusions	No data were excluded.
Replication	All attempts for replication were successful.
Randomization	Not relevant
Blinding	Not applicable.

Reporting for specific materials, systems and methods

We require information from authors about some types of materials, experimental systems and methods used in many studies. Here, indicate whether each material, system or method listed is relevant to your study. If you are not sure if a list item applies to your research, read the appropriate section before selecting a response.

Materials & experimental systems

n/a	Involved in the study
☒	☐ Antibodies
☒	☐ Eukaryotic cell lines
☒	☐ Palaeontology and archaeology
☐	☒ Animals and other organisms
☒	☐ Clinical data
☒	☐ Dual use research of concern

Methods

n/a	Involved in the study
☒	☐ ChIP-seq
☒	☐ Flow cytometry
☒	☐ MRI-based neuroimaging

Animals and other research organisms

Policy information about studies involving animals; ARRIVE guidelines recommended for reporting animal research, and Sex and Gender in Research

Laboratory animals	Laboratory animals were not used.
Wild animals	Wild animals were not used.
Reporting on sex	Only microbes were used.
Field-collected samples	The study did not involve field-collected samples.
Ethics oversight	No ethical approval was required as only microbes were used in this study in a lab context.

Note that full information on the approval of the study protocol must also be provided in the manuscript.
